# Gut microbiota in poultry: Roles in nutrition, metabolism and production performance

**DOI:** 10.1016/j.psj.2026.107644

**Published:** 2026-08-17

**Authors:** Zhuting Chen, Cheng Xin, Shuang Zhang, Wanyue Meng, Deming Zhang, Fei Zhang, Qinyi Zhan, Yanli Liu, Xin Yang, Xiaojun Yang, Zhouzheng Ren

**Affiliations:** a College of Animal Science and Technology, Northwest A&F University, Yangling, Shaanxi, 712100, China; b School of Life Sciences, Zhengzhou University, Henan, Zhengzhou, 450001, China

**Keywords:** Poultry, Gut microbiota, Nutrition, Metabolism, Production performance

## Abstract

The gut microbiota plays an important role in nutrient utilization, intestinal homeostasis, immune regulation, and production performance in poultry. Its composition and functional activity are shaped by complex interactions among host species, developmental stage, intestinal segment, diet, genetic background, and rearing environment. This review summarizes current knowledge of the poultry gut microbiota, with particular emphasis on its roles in nutrition, metabolism, and production performance. We first examine microbial composition and spatial distribution across major poultry species, including broiler chickens, laying hens, ducks, geese, and turkeys, and discuss the factors contributing to interspecies and interstudy variation. We then evaluate how dietary components, including fiber, protein, amino acids, and lipids, modulate microbial communities and metabolic pathways. Particular attention is given to microbial fermentation, short-chain fatty acid production, microbial nitrogen metabolism, bile acid transformation, and host–microbiota signaling, which collectively influence nutrient utilization, intestinal barrier function, immune homeostasis, and metabolic regulation. The potential of microbiota-targeted strategies, including probiotics, prebiotics, synbiotics, dietary fiber manipulation, reduced-protein diets, and emerging microbial interventions, to improve feed efficiency, growth performance, carcass characteristics, meat quality, and resilience to environmental stress is also discussed. In addition, the implications of gut microbiota regulation for nitrogen utilization, ammonia emissions, animal welfare, and antibiotic-free production are considered. Despite substantial progress, the translation of microbiome research into practical applications remains limited by inconsistent experimental designs, methodological heterogeneity, insufficient functional validation, and a lack of causal evidence. Integrating multi-omics approaches with controlled intervention studies and standardized experimental frameworks will be essential for defining causal microbiota–host interactions and developing precision microbiota-based strategies. Overall, a functional understanding of the diet–microbiota–host axis may provide new opportunities to improve poultry productivity, health, and sustainability.

## Highlights

• Gut microbiota integrates nutrition, metabolism, and production in poultry.
• Microbial metabolites regulate gut barrier and immune homeostasis.
• Diet shapes microbiota-mediated nutrient utilization and host physiology.
• Poultry microbiome research gaps and future priorities are identified.
• Microbiota-based strategies advance sustainable poultry production.

## Introduction

The gastrointestinal tract of poultry harbors a complex and dynamic microbial ecosystem that contributes to nutrient utilization, intestinal development, immune homeostasis, and resistance to enteric pathogens (Clavijo, et al., 2018; Pan, et al., 2014; Shang et al., 2018). The avian gastrointestinal tract is anatomically and physiologically specialized, comprising the crop, proventriculus, gizzard, small intestine, paired ceca, and cloaca, each of which provides distinct physicochemical conditions for microbial colonization (Oakley, et al., 2014b; Waite, et al., 2014). Consequently, poultry gut microbiota exhibit marked spatial and temporal heterogeneity, with microbial composition and function varying among species, intestinal segments, developmental stages, diets, and production systems (Kers, et al., 2018). As a key interface between dietary substrates and host physiology, the gut microbiota transforms otherwise indigestible nutrients into bioactive metabolites, including short-chain fatty acids (SCFAs), vitamins, amino acid derivatives, and bile acid metabolites, thereby contributing to host energy metabolism, intestinal homeostasis, and metabolic regulation (Koh, et al., 2016; Rowland, et al., 2018). Increasing evidence therefore indicates that gut microbial ecology is closely associated with nutrient utilization, feed efficiency, growth performance, and other economically important traits in poultry production (Oakley, et al., 2014b; Stanley, et al., 2013; Yan, et al., 2017).

The composition and function of the poultry gut microbiota are shaped by interacting host, dietary, environmental, and management factors (Kers, et al., 2018; Pan, et al., 2014). Differences among poultry species, including broiler chickens, laying hens, ducks, geese, and turkeys, are accompanied by distinct gastrointestinal physiology and microbial community structures (Waite, et al., 2014; Wei, et al., 2013). Microbial profiles also vary substantially among intestinal segments and change dynamically during post-hatch development (Lu, et al., 2003; Oakley, et al., 2014b). Diet composition, particularly the levels and sources of dietary fiber, protein, amino acids, and lipids, further influences microbial substrates, metabolic pathways, and metabolite production (Oakley, et al., 2014b), while housing conditions, heat stress, stocking density, water quality, and antimicrobial exposure can modify microbial stability and host–microbiota interactions (Kers, et al., 2018; Lara, et al., 2013). Although *Firmicutes* and *Bacteroidota* are frequently reported as major bacterial phyla in poultry, their relative abundance varies considerably according to species, age, intestinal location, diet, and experimental conditions (Oakley, et al., 2014b; Pan, et al., 2014; Wei, et al., 2013). Thus, the abundance of dominant bacterial taxa alone should not be considered a universal indicator of intestinal health. Instead, microbial functional capacity, metabolic activity, and host–microbiota interactions may provide more meaningful information for understanding nutrient utilization and production outcomes (Stanley, et al., 2013; Yan, et al., 2017).

With increasing restrictions on antibiotic growth promoters, microbiota-targeted nutritional strategies have received growing attention in poultry science (Gadde, et al., 2017). Probiotics, prebiotics, synbiotics, dietary fiber manipulation, polyunsaturated fatty acid supplementation, and reduced-protein diets have been investigated as approaches to modulate microbial communities, improve intestinal function, and enhance nutrient utilization (Yadav, et al., 2019). However, responses to these interventions are highly context-dependent and may vary with bird species, age, basal diet, ingredient composition, intervention dose, and rearing environment (Kers, et al., 2018; Yadav, et al., 2019). For example, the effects of dietary fiber depend on its physicochemical properties and inclusion level, whereas the outcomes of reduced-protein diets may depend on the degree of crude protein reduction, amino acid balance, and the availability of fermentable substrates for microbial nitrogen metabolism (Borda-Molina, et al., 2018; Jha, et al., 2021). Similarly, probiotic efficacy is influenced by strain identity, viability, dose, and interactions with the resident microbiota (Yadav, et al., 2019). These variations indicate that microbiota regulation cannot be reduced to a simple distinction between "beneficial" and "harmful" bacteria and instead requires a functional understanding of microbial communities and their metabolic interactions with the host (Diaz Carrasco, et al., 2019; Jha, et al., 2021; Yadav, et al., 2019). Emerging approaches, including fecal microbiota transplantation (FMT), bacteriophage-based interventions, and IgA-mediated microbial regulation, further expand the potential for targeted manipulation of poultry gut ecosystems (Diaz Carrasco, et al., 2019).

The nutritional importance of the gut microbiota is closely linked to its capacity to convert dietary substrates into metabolites that influence host physiology (Pan, et al., 2014). Microbial fermentation of dietary fiber produces SCFAs, including acetate, propionate, and butyrate, which contribute to epithelial energy supply, intestinal barrier function, and host metabolic regulation (Jha, et al., 2021; Koh, et al., 2016). These metabolites can also act as signaling molecules through pathways involving G protein-coupled receptors such as GPR41 (FFAR3) and GPR43 (FFAR2), thereby influencing epithelial, immune, and metabolic processes (Morrison, et al., 2016). Microbial metabolism of proteins and amino acids, in contrast, can generate ammonia and other metabolites when fermentable nitrogen exceeds host requirements, potentially affecting intestinal health, nitrogen utilization, and environmental nitrogen losses (Jha, et al., 2021). Microbial transformation of bile acids and interactions with host immune signaling further illustrate the interconnected nature of diet–microbiota–host communication (Koh, et al., 2016). Specifically, microbial bile acid metabolism can influence host physiology through bile acid-responsive receptors, including farnesoid X receptor (FXR) and Takeda G protein-coupled receptor 5 (TGR5) (Fiorucci, et al., 2009). Intestinal bacteria expressing bile salt hydrolase (BSH) activity participate in bile acid deconjugation and modification, thereby altering the composition and signaling activity of microbial-derived bile acid metabolites (Begley, et al., 2006). FXR activation contributes to the regulation of bile acid homeostasis, lipid metabolism, and intestinal barrier maintenance, whereas TGR5 signaling is involved in energy metabolism and inflammatory regulation (Fiorucci, et al., 2009). In poultry, dietary composition and microbial community structure may influence bile acid transformation processes, providing a potential mechanistic link between microbiota regulation, nutrient utilization, immune homeostasis, and production performance (Yin, et al., 2010). However, the specific microbial taxa responsible for regulating bile acid signaling and the causal relationship between microbial bile acid metabolism and poultry production traits require further investigation (Clavijo, et al., 2018). These processes provide potential mechanistic links between microbial activity and economically relevant outcomes, including feed efficiency, growth, carcass characteristics, meat quality, and resilience to environmental stress (Stanley, et al., 2014). In addition to metabolic regulation, gut microbiota plays a critical role in shaping intestinal immune responses through microbial recognition pathways (Clavijo, et al., 2018). Microbial-associated molecular patterns (MAMPs) derived from intestinal bacteria can activate pattern recognition receptors (PRRs), including Toll-like receptors (TLRs), thereby regulating downstream inflammatory signaling pathways such as nuclear factor-κB (NF-κB) (Kogut, et al., 2016). Excessive activation of these pathways may increase the production of pro-inflammatory cytokines, including interleukin-1β (IL-1β), interleukin-6 (IL-6), and tumor necrosis factor-α (TNF-α), resulting in impaired intestinal homeostasis and reduced nutrient utilization efficiency (Kogut, et al., 2016). Conversely, beneficial microbial communities and microbial-derived metabolites can maintain immune balance by supporting epithelial integrity and preventing excessive inflammatory activation (Pan, et al., 2014).

Despite rapid advances in poultry microbiome research, important knowledge gaps remain. Many studies are observational and identify associations between microbial taxa and production traits without establishing causal relationships (Oakley, et al., 2014b). Moreover, differences in experimental design, bird breed and age, dietary formulation, sampling location, sample collection and storage, sequencing platforms, and bioinformatic pipelines can contribute to variation among studies and complicate cross-study comparisons (Kers, et al., 2018). Although 16S rRNA gene sequencing has substantially advanced the characterization of microbial community composition, its limited taxonomic and functional resolution constrains strain-level and pathway-level interpretation (Johnson, et al., 2019). Shotgun metagenomics provides broader information on microbial functional potential but does not necessarily reflect active gene expression or metabolite production (Franzosa, et al., 2019). Integrating metagenomics with metabolomics, metatranscriptomics, proteomics, and host transcriptomics, together with controlled intervention studies, will therefore be essential for establishing causal relationships between microbial functions and poultry phenotypes (Zierer, et al., 2018).

This review focuses specifically on the roles of gut microbiota in poultry nutrition, metabolism, and production performance. We first examine the composition and spatial distribution of gut microbiota across major poultry species and gastrointestinal segments, with consideration of developmental stage and experimental context. We then discuss how dietary components and nutritional interventions shape microbial composition and function, with particular emphasis on fiber, protein and amino acid metabolism, microbial metabolites, and host–microbiota signaling. The mechanistic links between microbiota regulation and nutrient utilization, intestinal integrity, immune homeostasis, and production performance are further evaluated. Finally, we consider the practical and translational implications of microbiota-targeted strategies for improving feed efficiency, carcass and meat quality, heat-stress resilience, nitrogen utilization, ammonia emissions, animal welfare, and antibiotic-free production. By integrating microbial ecology, nutritional metabolism, host physiology, and production outcomes, this review aims to provide a poultry-specific framework for understanding functional microbiota regulation and to identify priorities for precision and sustainable poultry production.

## Composition, spatial distribution, and functional characteristics of the poultry gut microbiota

The gut microbiota of poultry exhibits distinct spatial, temporal, and diet-dependent characteristics that reflect differences in gastrointestinal anatomy, feeding behavior, and nutrient utilization (Oakley, et al., 2014b; Waite, et al., 2014). Compared with the original emphasis on poultry, the present review focuses primarily on poultry, in which microbial communities differ substantially among broilers, layers, ducks, geese, and turkeys and across intestinal segments (Waite, et al., 2014; Wei, et al., 2013). These differences are biologically important because the microbial ecosystem of the crop, ileum, ceca, and other gastrointestinal compartments is exposed to distinct pH values, oxygen availability, digesta transit rates, and nutrient substrates (Oakley, et al., 2014b). Despite these variations, multiple species harbor highly complex microbial ecosystems dominated by bacteria, with *Firmicutes* and *Bacteroidetes* representing the two most abundant phyla in the mature intestine (Oakley, et al., 2014b; Wei, et al., 2013). However, their relative abundance varies substantially among species, ages, intestinal sites, diets, and analytical methods, and therefore should not be regarded as a universal microbiological signature of healthy poultry (Waite, et al., 2014). The relative proportions and functional potential of these microbial communities are closely linked to dietary composition and play a decisive role in nutrient metabolism, intestinal health, and production performance (Pan, et al., 2014; Stanley, et al., 2014). Detailed information on major microbial taxa and their functions is provided in Table 1.

**Table 1:** Composition and functional characteristics of the gut microbiota in poultry.

Poultry species	Age/production stage	Intestinal segment	Dominant microbial taxa	Relative abundance of major phyla	Major microbial functions	Sequencing approach	Key references
Broiler chickens	Starter, grower, and finisher stages	Duodenum/Ileum/Cecum	Lactobacillus, *Firmicutes*-affiliated taxa; *Bacteroidetes* dominant in cecum	*Firmicutes* predominated in the small intestine, whereas *Bacteroidetes* became increasingly abundant in the cecum	Competitive exclusion of pathogens; energy saving translating into improved productive performance; nutrient metabolism; immune homeostasis	16S rRNA sequencing; QMP (quantitative microbiome profiling); metagenomics	(Feng, et al., 2023); (Oakley, et al., 2014a); (Stanley, et al., 2014)
Laying hens	Pullet (pre-laying) and laying stages	Ileum/Cecum	Phocaeicola, Bacteroides (most abundant genera in Jianghan chicken); Age-associated enrichment of Alistipes	*Firmicutes* predominated during early development, whereas *Bacteroidetes* increased during the laying period	Gut immune function; nutrient absorption; fiber degradation increasing with age; support of laying performance	16S rRNA sequencing; MAGs (metagenome-assembled genomes)	(Shen, et al., 2024); (Elokil, et al., 2020)
Ducks	Growing and adult stages	Duodenum/Ileum/Cecum	*Lactobacillus, Bacteroides, Escherichia-Shigella, Enterococcus*, and *Firmicutes*-affiliated taxa	*Firmicutes* predominated in the ileum, whereas *Bacteroidetes* predominated in the cecum	SCFA production; carbohydrate fermentation; intestinal energy metabolism	16S rRNA sequencing	(Wang, et al., 2018); (Yang, et al., 2020); (Best, et al., 2016); (He, et al., 2019)
Geese	Growing and adult stages	Cecum (7 GI segments profiled)	*Bacteroides, Faecalibacterium*, and Butyricicoccus	*Bacteroidetes* and *Firmicutes* were predominant phyla	Fiber fermentation; carbohydrate metabolism; SCFA production; host–microbiome interactions	16S rRNA sequencing; whole-genome sequencing combined with gut metagenomic analysis	(Yang, et al., 2018); (Li, et al., 2022); (Yu, et al., 2021);
Turkeys	Growing and finishing stages	Small intestine/Cecum/Cloaca	*Lactobacillus, Streptococcus*, and *Clostridium* XI	*Firmicutes* were the predominant phylum throughout the gastrointestinal tract, whereas *Bacteroidetes* were relatively enriched in the cecum	Resistance to pathogen infection; nutrient metabolism; intestinal maturation; contribution to optimal growth	16S rRNA sequencing (Ion Torrent); pyrosequencing-based metagenomics	(Taylor, et al., 2020); (Wei, et al., 2016); (D'Andreano, et al., 2017); (Rzeznitzeck, et al., 2022); (Scupham, 2007)

### Spatial distribution and species-specific differences

The poultry gastrointestinal tract is characterized by pronounced spatial heterogeneity in microbial composition and function (Oakley, et al., 2014b). The crop, proventriculus, gizzard, small intestine, paired ceca, and cloaca differ in pH, oxygen availability, nutrient accessibility, transit time, and host-derived substrates, thereby creating distinct ecological niches for microbial colonization (Oakley, et al., 2014b; Waite, et al., 2014). Consequently, microbial communities vary substantially along the gastrointestinal tract, and the functional significance of a given microbial taxon depends strongly on its anatomical location and local environmental context (Oakley, et al., 2014b).

Microbial communities in the small intestine are generally exposed to more readily available dietary substrates and host-derived nutrients, whereas the ceca provide a relatively stable environment for the fermentation of complex carbohydrates and the production of microbial metabolites (Oakley, et al., 2014b; Pan, et al., 2014). These spatial differences have important implications for microbiome research. For example, a dietary intervention that increases cecal SCFA production may not produce the same microbial or metabolic response in the ileum, where nutrient absorption, digesta flow, and bile acid exposure impose different selective pressures (Oakley, et al., 2014b). Therefore, sampling location should be considered a critical experimental variable when evaluating associations between gut microbiota, nutrient metabolism, and production performance (Kers, et al., 2018). Measurements based solely on fecal or cecal samples may not fully represent microbial processes occurring in the upper or small intestinal tract (Oakley, et al., 2014b).

Species-specific differences further complicate the spatial organization of the poultry gut microbiota (Waite, et al., 2014). Broiler chickens have been extensively studied because of their rapid growth and intensive nutrient utilization, whereas laying hens experience prolonged production cycles characterized by sustained nutrient demands associated with egg production and mineral metabolism (Pan, et al., 2014). Ducks and geese possess distinct digestive physiology and feeding behaviors, while turkeys differ from chickens in body size, growth trajectory, dietary requirements, and susceptibility to enteric challenges (Waite, et al., 2014). These biological differences may contribute to species-specific patterns of microbial colonization and metabolic activity (Wei, et al., 2013). Studies in ducks have demonstrated substantial regional and temporal variation in microbial communities during intestinal development and under different rearing conditions, whereas studies in laying hens have revealed dynamic microbial changes across major gastrointestinal sections (Waite, et al., 2014). Thus, microbial biomarkers or nutritional interventions identified in broiler chickens should not be assumed to have equivalent biological effects in layers, ducks, geese, or turkeys without species-specific validation (Kers, et al., 2018).

Spatial heterogeneity also has direct implications for interpreting production-related phenotypes (Oakley, et al., 2014b; Stanley, et al., 2014). Microbial functions in the small intestine may influence nutrient digestion and absorption more directly, whereas cecal microbial activity may contribute more substantially to carbohydrate fermentation, SCFA production, nitrogen metabolism, and mucosal immune regulation (Jha, et al., 2021; Oakley, et al., 2014b). A comprehensive assessment of microbiota-mediated effects on poultry performance should therefore integrate microbial measurements from anatomically relevant intestinal sites with host physiological and production data (Kers, et al., 2018; Waite, et al., 2014). Such an approach is particularly important when evaluating dietary interventions, because microbial responses observed in one intestinal segment may not reflect changes in other regions of the gastrointestinal tract (Kers, et al., 2018; Oakley, et al., 2014b).

### Major microbial taxa and functional associations

At the phylum level, *Firmicutes* and *Bacteroidota* are commonly reported as major components of the gut microbiota in poultry, although their relative abundance varies according to species, age, intestinal segment, diet, host genotype, rearing environment, and sequencing methodology (Bajagai, et al., 2024). Members of *Firmicutes*, including *Lactobacillus, Clostridium, Ruminococcus*, and *Faecalibacterium*, are involved in carbohydrate fermentation and the production of SCFAs and other microbial metabolites (He, et al., 2025, 2023). Bacteroidota, including *Bacteroides* and related polysaccharide-degrading taxa, possess extensive enzymatic capacities for the degradation of complex carbohydrates and non-starch polysaccharides, thereby contributing to substrate utilization and microbial fermentation (Bajagai, et al., 2024; Ma, et al., 2024; Wang, et al., 2023).

However, taxonomic composition alone provides an incomplete representation of microbial function. Bacterial strains within the same genus may differ substantially in metabolic capacity, and the physiological consequences of microbial changes depend on substrate availability, intestinal location, host physiology, and interactions among microbial populations (He, et al., 2025). Therefore, associations between the relative abundance of *Firmicutes* or *Bacteroidota* and production traits such as feed efficiency should be interpreted cautiously unless supported by functional evidence from metagenomics, metatranscriptomics, metabolomics, or controlled intervention studies.

The same principle applies to potentially opportunistic microbial groups. *Proteobacteria* are generally detected at relatively low abundance in healthy poultry but may increase under conditions of dietary imbalance, environmental stress, or pathogen challenge (Zhao, et al., 2025). Nevertheless, *Proteobacteria* should not be considered inherently pathogenic because this phylum contains both commensal and opportunistic organisms (Ma, et al., 2024). Their biological significance should instead be evaluated in the context of microbial function, pathogen burden, host inflammatory status, and intestinal barrier integrity. Accordingly, the identification of a microbial taxon as a biomarker of health or production performance requires functional and physiological validation rather than reliance on taxonomic abundance alone.

Species-specific production objectives may further influence the biological interpretation of microbial signatures. Microbial features associated with rapid growth and feed efficiency in broilers may not have the same functional significance in laying hens, where long-term egg production and mineral metabolism are major physiological priorities (Bajagai, et al., 2024). Similarly, microbial biomarkers identified in chickens may not be directly applicable to ducks, geese, or turkeys because of differences in digestive physiology, feeding behavior, growth trajectory, and production systems. Future studies should therefore prioritize species-specific microbial functions and metabolic pathways rather than assuming the existence of a universal "core microbiota" across poultry species.

### Developmental dynamics across age

Gut microbiota establishment begins immediately after hatch and represents a critical developmental process that influences intestinal maturation, immune development, and metabolic capacity. During the first days of life, microbial colonization is shaped by hatchery conditions, environmental and maternal microbial exposure, feed and water, housing systems, and antimicrobial use. Early microbial communities are generally less stable than those of mature birds, and this period may represent a window of increased susceptibility to nutritional stress and enteric disorders (Bajagai, et al., 2024).

In chickens, microbial succession involves rapid changes during the early post-hatch period, followed by progressive stabilization as the intestinal ecosystem matures (Bajagai, et al., 2024). However, the timing and trajectory of microbial development differ among broilers, layers, ducks, geese, and turkeys and are further influenced by growth rate, dietary formulation, housing conditions, and disease exposure (Borda-Molina, et al., 2018; Pan, et al., 2014). Age should therefore be considered a major biological variable when comparing microbiome datasets or assessing the efficacy of microbiota-targeted nutritional interventions.

Early nutritional interventions, including functional carbohydrates, probiotics, and other microbiota-targeted strategies, have been investigated for their potential to influence microbial maturation and intestinal development (Gaggia, et al., 2010). Early-life microbiota transplantation has also been explored as a strategy for shaping long-term microbial establishment and potentially influencing growth and intestinal health (van der Eijk, et al., 2020). However, the effectiveness and safety of such interventions require careful evaluation, particularly with respect to donor selection, microbial consistency, pathogen transmission, and long-term outcomes. Thus, early-life microbiota modulation represents a promising but still developing strategy that requires standardized protocols and species-specific validation before broad commercial application.

Importantly, microbial maturation should not be equated with an increase in taxonomic diversity alone. A more diverse microbial community is not necessarily more functionally mature or beneficial, whereas a community with lower taxonomic diversity but greater functional redundancy may provide enhanced ecosystem stability (Sergeant, et al., 2014). Therefore, age-related microbial development should be evaluated by integrating taxonomic composition with functional metagenomics, metabolomics, and host physiological measurements to determine whether microbial succession corresponds to meaningful functional maturation.

### Functional diversity and metabolic capacity

Beyond taxonomic composition, the functional capacity of the gut microbiota is a critical determinant of its nutritional and physiological significance. Microbial genes involved in carbohydrate degradation, amino acid metabolism, bile acid transformation, vitamin biosynthesis, and other metabolic pathways collectively expand the biochemical capacity of the gastrointestinal ecosystem and influence the availability of nutrients and bioactive metabolites to the host (Krautkramer, et al., 2021).

The functional contribution of the microbiota is determined not only by the presence of specific microbial taxa but also by interactions among microorganisms and the substrates available within the intestinal environment. Cross-feeding among microbial populations can facilitate the conversion of complex dietary components into metabolites that would otherwise be inaccessible to the host. Similarly, microbial metabolic pathways may respond dynamically to changes in diet, age, intestinal location, and environmental conditions (Zhao, et al., 2013). Consequently, microbial functions cannot be reliably inferred from taxonomic abundance alone.

This functional perspective is particularly relevant to poultry production because microbial activity may influence nutrient availability, intestinal barrier function, immune homeostasis, and metabolic efficiency without necessarily causing large changes in overall community composition. Integrating microbial taxonomic profiles with metagenomic, metatranscriptomic, metabolomic, and host physiological data may therefore provide a more comprehensive understanding of how gut microbial functions contribute to poultry nutrition and production performance. Such an integrative approach also provides a foundation for identifying functional biomarkers and developing precision microbiota-targeted strategies tailored to specific poultry species, production stages, and dietary conditions.

## Factors influencing the composition and function of poultry gut microbiota

**Fig. 1:**
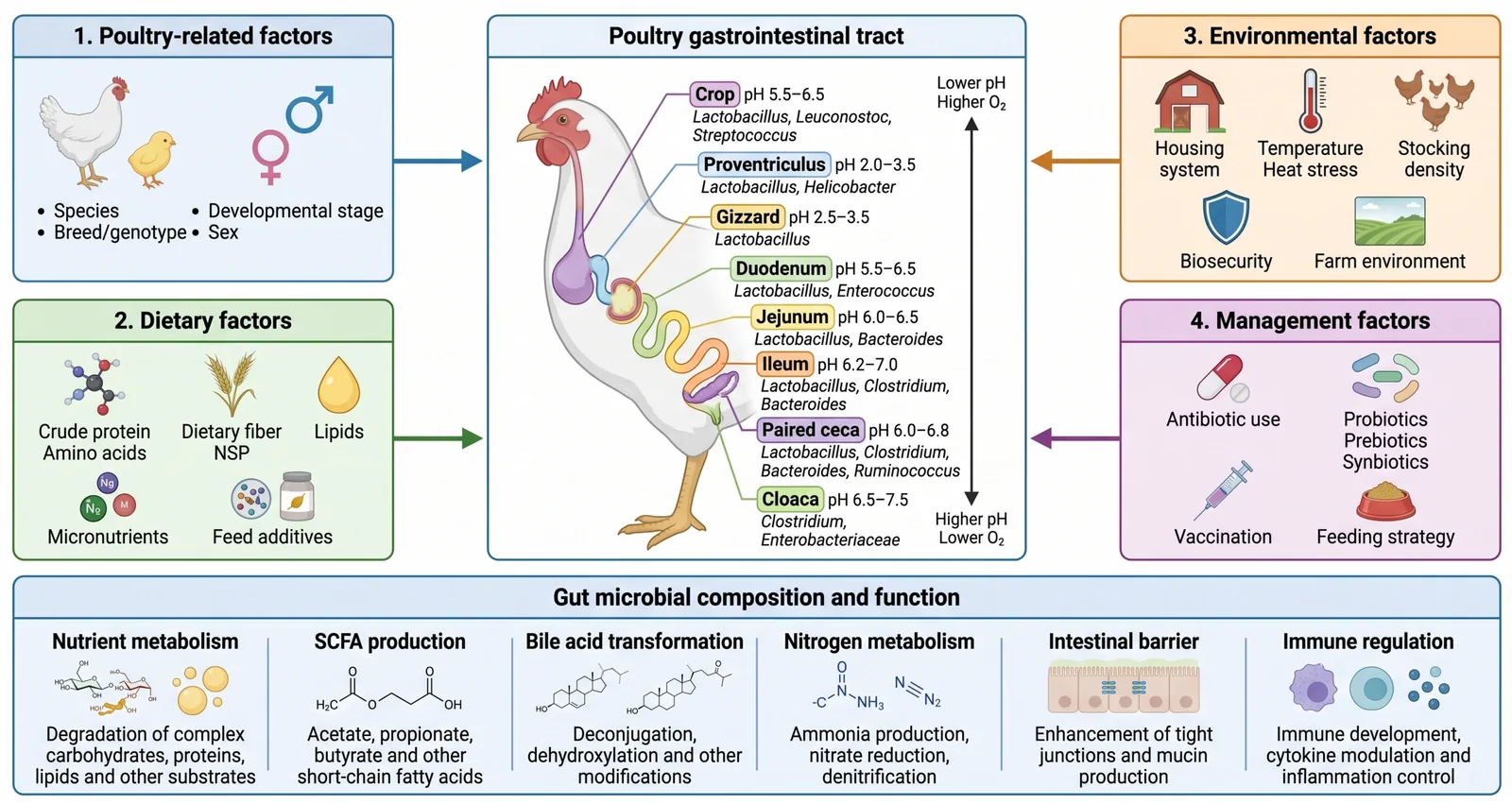
**Overview of the gut microbiota ecosystem in poultry and its major determinants.** The composition and functional activity of the poultry gut microbiota are shaped by complex interactions among host-related, dietary, environmental, and management factors. Poultry species, genetic background, age, and physiological status influence microbial colonization and ecosystem development, whereas dietary fiber, protein and amino acid balance, lipids, micronutrients, and feed additives regulate microbial substrate availability and metabolic activity. Environmental and management factors, including housing conditions, stocking density, heat stress, water quality, litter quality, and antimicrobial exposure, further affect microbial community structure and stability. The resulting microbial composition, functional capacity, and metabolite production interact with host nutrient metabolism, intestinal barrier integrity, immune homeostasis, and energy metabolism, ultimately influencing nutrient utilization, growth performance, feed efficiency, carcass traits, meat quality, disease resistance, animal welfare, and environmental sustainability.

The gut microbiota of poultry is shaped by complex interactions among dietary, host, environmental, and management factors (Kers, et al., 2018). Among these factors, diet is one of the most powerful and readily modifiable determinants of microbial composition and function (Pan, et al., 2014). However, dietary effects are not independent of poultry species, age, gastrointestinal segment, genetic background, housing conditions, and environmental exposure. Therefore, the effects of nutritional interventions should be interpreted within the broader biological and production context (Fig. 1; Table 2).

**Table 2:** Major nutritional, host, environmental, and management factors influencing the poultry gut microbiota.

Category	Factor	Specific variables	Effects on gut microbiota	Functional consequences	Production relevance	Key references
Dietary	Dietary fiber	NSPs (arabinoxylans, β-glucans), cellulose, resistant starch	Enhances carbohydrate-fermenting and SCFA-producing microbial populations (e.g., *Lactobacillus* and members of Ruminococcaceae/Lachnospiraceae); soluble NSPs increase cecal SCFA production	SCFA production (acetate, butyrate, propionate); microbial cross-feeding; modulation of gut immunity	Nutrient utilization; FCR improvement	(Jha, et al., 2021); (Singh et al., 2021); (Nguyen, et al., 2021)
Dietary	Protein	Crude protein level; protein source; digestibility	Alters proteolytic bacterial populations; excessive or poorly digestible protein promotes hindgut proteolytic fermentation and increases production of ammonia, H₂S, phenols, and indoles	Nitrogen metabolism; production of ammonia and other protein fermentation metabolites; increased risk of wet litter	Nitrogen efficiency; emissions; intestinal health	(Zhao, et al., 2019); (Gilbert, et al., 2018); (J. Apajalahti, 2016)
Dietary	Amino acids	Essential amino acid supplementation (lysine, methionine, threonine, and tryptophan)	Influences microbial nitrogen availability and amino acid metabolism; modulates the abundance of amino acid-utilizing microorganisms	Microbial–host nutrient competition; immune and metabolic regulation	Growth performance; feed efficiency	(J. Apajalahti, 2016)
Dietary	Feed additives (alternatives to antibiotics)	Probiotics, prebiotics, organic acids, exogenous enzymes	Modulate gut microbiota composition toward beneficial configurations; organic acids restore dysbiosis under stress	Improved nutrient digestion and absorption; enhanced intestinal barrier function and immune homeostasis	Growth; FCR; carcass yield; antibiotic-free production	(Yadav, et al., 2019); (Kogut, 2019); (Dai, et al., 2022)
Host	Species	Chicken, duck, goose, turkey	Species-specific microbial composition; broiler chickens and turkeys harbor distinct intestinal bacteriomes despite same-region similarity	Differential metabolic capacity; different dietary interventions needed for bacteriome modulation	Production specialization	(Feng, et al., 2023); (Oakley, et al., 2014a); (Wei, et al., 2016)
Host	Age	Post-hatch to adult	Microbial diversity increases rapidly during early post-hatch development; colonization patterns differ between layer- and meat-type chickens, followed by progressive community stabilization	Functional maturation; segment-specific community formation	Lifetime productivity	(Kers, et al., 2018); (Feng, et al., 2023)
Host	Genetics	Breed/genetic line (layer vs. meat-type)	Host-specific microbial colonization; colonization patterns differ between breeds	Host–microbiota interactions; varied microbial diversity across genetic lines	Feed efficiency improvement; genetic selection for production traits	(Kers, et al., 2018)
Environment	Heat stress	High ambient temperature and humidity	Induces gut dysbiosis, reduces microbial stability, and alters the abundance of beneficial and opportunistic bacterial populations	Barrier dysfunction (downregulated tight junction proteins); intestinal inflammation	Reduced growth performance; heat-stress resilience	(Patra, et al., 2021); (Liu, et al., 2022)
Management	Housing	Cage, floor (litter/mesh), free-range	Housing systems influence microbial diversity and composition through differences in environmental exposure, diet, and microbial transmission; litter and mesh-floor systems may alter dominant microbial taxa	Microbial ecosystem structure; microbial exposure from environment shapes colonization	Health, welfare, and productivity	(Hubert, et al., 2019); (Wang, et al., 2018); (Kers, et al., 2019)
Management	Stocking density	Normal vs. high density	High stocking density (HSD) decreases *Bacteroidetes* abundance; disturbs balance of microbial ecosystems; increases competitive and exploitative interactions	Intestinal barrier dysfunction; inflammation (↑IL-1β, TNF-α); compromised growth	Disease risk; growth performance loss	(Dai, et al., 2022); (Li, et al., 2023)
Management	Water quality	pH, mineral content, acidification	Acidified drinking water modifies gastrointestinal microbiota; improves intestinal barrier integrity	Improved intestinal barrier integrity and nutrient utilization	FCR improvement; production consistency	(Zhang, et al., 2022); (Hamid, et al., 2018)
Management	Antimicrobial exposure	Antibiotics (AGPs and therapeutics)	Disrupts microbiota; intensive antibiotic use shifts cecal microbiota toward antibiotic-resistant bacteria; marked enrichment of resistome	Dysbiosis; antibiotic resistance gene dissemination; loss of colonization resistance	Antibiotic stewardship; food safety	(Gadde, et al., 2017); (Mancabelli, 2016); (Yadav, et al., 2019)

### Dietary factors

Dietary macronutrients provide the principal substrates available to intestinal microorganisms and therefore strongly influence microbial ecology, metabolic activity, and host nutrient utilization. The quantity, chemical characteristics, and digestibility of dietary carbohydrates, proteins, and lipids determine which microbial populations are favored and which metabolic pathways are activated (Leeming, et al., 2019; Scott, et al., 2013).

#### Macronutrients and fermentable substrates

Dietary fiber, particularly non-starch polysaccharides (NSP), is a major substrate for microbial fermentation in the poultry gastrointestinal tract. However, its effects on gut microbiota and production performance depend strongly on fiber source, chemical structure, solubility, viscosity, particle size, inclusion level, enzyme supplementation, bird age, and basal diet composition (Jha, et al., 2021). Soluble and readily fermentable fibers may selectively support carbohydrate-fermenting microorganisms and increase SCFA production, whereas excessive amounts of poorly digestible or highly viscous fiber may impair nutrient digestibility, alter digesta passage, and compromise performance (Jha, et al., 2021).

Microbial degradation of complex carbohydrates generates SCFAs, including acetate, propionate, and butyrate, which can contribute to epithelial energy metabolism, intestinal barrier integrity, and immune regulation (Flint, et al., 2012). These metabolites also act as signaling molecules through pathways involving GPR41 (FFAR3) and GPR43 (FFAR2) (Mann, et al., 2024). Butyrate may further support epithelial integrity by regulating tight-junction proteins, including zonula occludens-1 (ZO-1), occludin, and claudins (Moquet, 2016). Thus, the nutritional effects of dietary fiber may arise from both enhanced microbial fermentation and downstream host signaling.

Nevertheless, responses to dietary fiber are not consistent across poultry studies. Differences in fiber physicochemical properties, inclusion levels, bird age, genetic background, basal diet, environmental conditions, and experimental methodology may contribute to divergent microbial and production outcomes (Jha, et al., 2021). Therefore, dietary fiber should not be evaluated simply according to crude fiber concentration. Instead, its fermentability, viscosity, microbial accessibility, and capacity to generate beneficial metabolites should be considered when designing species- and age-specific feeding strategies.

#### Protein availability and microbial nitrogen metabolism

Dietary protein is a major determinant of gut microbial composition and metabolic activity, particularly when the amount of protein escaping digestion in the small intestine increases. Undigested proteins and amino acids reaching the distal intestine can serve as substrates for microbial proteolysis, generating ammonia, phenols, indoles, amines, and branched-chain fatty acids (BCFAs). Some microbial amino acid metabolites may have physiological functions, whereas excessive production of ammonia and other potentially harmful compounds may adversely affect intestinal integrity and host metabolism (Rist, et al., 2013).

The relationship between dietary protein and gut microbiota depends on both crude protein concentration and amino acid balance. Reducing dietary crude protein while maintaining adequate levels of essential amino acids can decrease the flow of undigested protein to the distal intestine and reduce excessive proteolytic fermentation. However, the effects of protein reduction are not linear. Excessive protein restriction or inadequate amino acid supplementation may impair growth, nutrient utilization, immune function, and production performance (Kumar, et al., 2016; van Harn, et al., 2019). Therefore, precision protein nutrition should focus on optimizing protein and amino acid supply rather than simply minimizing dietary crude protein concentration.

The specific amino acid profile may also influence microbial metabolism. Amino acids such as lysine, methionine, threonine, and tryptophan contribute not only to host protein synthesis but also to microbial nitrogen availability and the production of bioactive metabolites. Threonine is particularly relevant to intestinal mucin synthesis and barrier maintenance, whereas tryptophan-derived microbial metabolites may influence intestinal and immune signaling (Kim, et al., 2024; Le Floc'h, et al., 2011; Lee, et al., 2020). Thus, the effects of amino acid nutrition on the gut microbiota should be considered in relation to amino acid digestibility, microbial substrate availability, and host requirements.

From a production perspective, optimizing dietary protein and amino acid utilization may simultaneously improve feed efficiency and reduce nitrogen excretion. Reduced nitrogen availability for excessive microbial proteolysis may decrease ammonia production and potentially lower environmental nitrogen losses. However, the quantitative contribution of intestinal microbial nitrogen metabolism to total farm-level ammonia emissions remains insufficiently defined and requires integration of dietary nitrogen balance, microbial metabolomics, manure microbiology, housing conditions, and direct emission measurements.

#### Lipids and lipid-derived metabolites

Dietary lipids influence the gut microbiota through changes in microbial membrane metabolism, bile acid metabolism, energy availability, and host inflammatory signaling (Jadhav, et al., 2024). The effects depend on fatty acid composition, chain length, degree of saturation, and inclusion level. Polyunsaturated fatty acids (PUFA), particularly n-3 PUFA, may modulate microbial community structure and inflammatory responses and may contribute to intestinal barrier maintenance, whereas excessive dietary saturated fat may adversely affect microbial balance and metabolic homeostasis (Cherian, 2015).

Dietary lipids may also influence the intestinal bile acid pool, thereby affecting host–microbiota interactions and metabolic signaling. Because bile acids function as both digestive molecules and signaling mediators, lipid nutrition may indirectly affect microbial ecology through changes in bile acid availability and transformation. These interactions may have implications for energy metabolism, intestinal function, and meat quality(Hu, et al., 2024) . However, the effects of specific lipid sources on poultry gut microbiota remain less well characterized than those of dietary carbohydrates and protein, and further poultry-specific studies are needed.

### Micronutrients and feed additives

Micronutrients, including zinc, selenium, iron, and vitamins A and E, can influence gut microbial ecology through interactions with microbial growth, intestinal barrier integrity, oxidative status, and host immune function. Zinc and selenium, for example, may support intestinal barrier function and antioxidant capacity, whereas vitamins A and E contribute to mucosal immunity and protection against oxidative stress (Alagawany, et al., 2020; Surai, et al., 2018). However, the effects of micronutrient supplementation on microbial composition are highly dependent on dose, chemical form, baseline nutritional status, and bird age. Excessive supplementation may also alter microbial ecology in unintended ways (Adedokun, et al., 2018). Therefore, micronutrient–microbiota interactions should be evaluated within the context of overall dietary formulation rather than as isolated effects.

Feed additives provide additional opportunities to regulate microbial communities. Probiotics, prebiotics, synbiotics, phytogenic compounds, organic acids, and exogenous enzymes may alter microbial composition or metabolic activity and thereby influence intestinal function and nutrient utilization. However, these interventions are discussed separately in Section 4 because their effects depend on specific mechanisms of action, microbial targets, dosage, and practical application.

### Host-related factors

Dietary effects on the gut microbiota are strongly modified by host conditions (Kers, et al., 2018). Poultry species and genetic background influence gastrointestinal physiology, immune responses, nutrient requirements, and microbial colonization (Singh, et al., 2012). Age is particularly important because microbial communities undergo rapid succession during early post-hatch development before gradually stabilizing as the gastrointestinal ecosystem matures (Lu, et al., 2003). Therefore, the same nutritional intervention may produce different microbial and production responses at different developmental stages.

### Environmental and management factors

Environmental and management factors can further modify gut microbial communities (Deusch, et al., 2015). Housing systems, stocking density, litter quality, water quality, temperature, humidity, and exposure to pathogens or antimicrobials may alter microbial transmission, community stability, and host susceptibility to dysbiosis (Kers, et al., 2018). Heat stress is particularly relevant to intensive poultry production because it can reduce feed intake, disrupt intestinal barrier integrity, alter microbial composition, and increase inflammatory and oxidative stress (Sohail, et al., 2010). Microbiota-targeted nutritional strategies may partially mitigate these effects, but their efficacy depends on bird age, genotype, environmental conditions, and baseline microbial status.

Water quality is another potentially important but underappreciated determinant of poultry gut microbiota. Water pH, mineral composition, microbial contamination, and biofilm formation may influence microbial exposure and intestinal stability (Kers, et al., 2018). Similarly, antimicrobial use can cause substantial and persistent changes in microbial community structure and may contribute to antimicrobial resistance (Lin, et al., 2013). These factors should therefore be considered when interpreting microbiome data and evaluating microbiota-targeted interventions under commercial conditions.

### Methodological sources of variation

In addition to biological factors, substantial variation among poultry microbiome studies can arise from differences in experimental design and analytical methodology. Breed and age, dietary formulation, housing conditions, sampling location, sample collection and storage, DNA extraction procedures, primer selection, sequencing platforms, and bioinformatic pipelines may all influence the microbial profiles obtained (Kers, et al., 2018). Differences in sampling sites are particularly important because microbial communities vary substantially along the gastrointestinal tract, and fecal or cecal samples may not accurately represent microbial processes occurring in the small intestine (Stanley, et al., 2013).

The choice of sequencing method also affects the interpretation of microbial ecology. 16S rRNA gene sequencing is widely used to characterize bacterial community composition but provides limited resolution at the strain level and offers only indirect information about functional potential (Oakley, et al., 2014b). Shotgun metagenomics provides broader information on microbial genes and metabolic pathways but does not necessarily indicate active gene expression or metabolite production (Sergeant, et al., 2014). Moreover, functional predictions based solely on taxonomic profiles should be interpreted cautiously because the presence of microbial genes does not necessarily indicate their expression or physiological activity.

Therefore, comparisons among poultry microbiome studies should account for both biological and methodological sources of variation. Standardized experimental designs, consistent sampling strategies, appropriate controls, and integrated multi-omics approaches will be essential for improving reproducibility and identifying robust microbial biomarkers. These considerations are particularly important when translating microbiome associations into nutritional interventions intended for commercial poultry production.

## Functional roles of poultry gut microbiota in nutrient metabolism and production performance

**Fig. 2:**
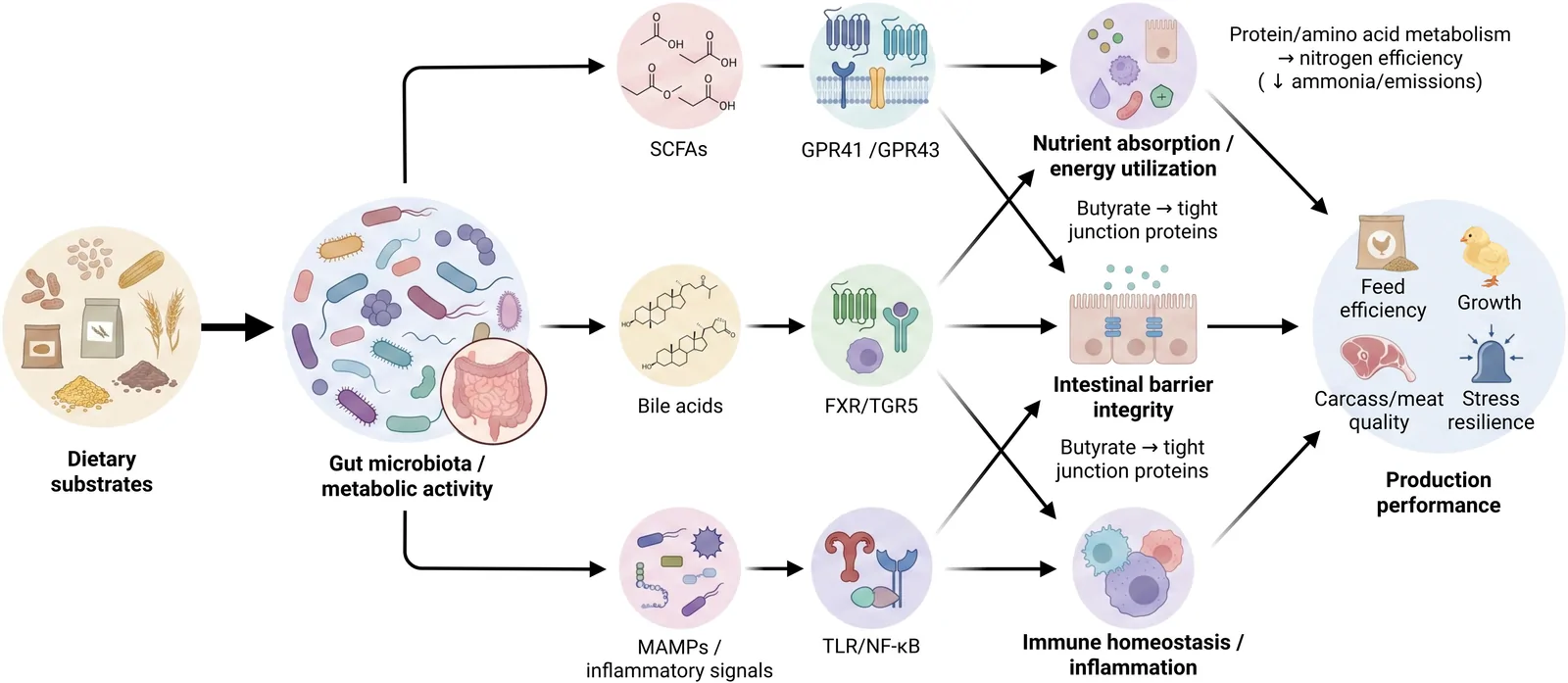
**Mechanistic pathways linking gut microbiota to nutrient metabolism and production performance in poultry.** Dietary substrates shape gut microbial composition and functional activity, which in turn generate microbial metabolites that influence host physiology through multiple signaling pathways. Fermentation of dietary carbohydrates and fiber produces short-chain fatty acids (SCFAs), including acetate, propionate, and butyrate, which can activate GPR41 and GPR43 and regulate energy metabolism, immune responses, and epithelial homeostasis. Butyrate may further promote intestinal barrier integrity by regulating tight-junction proteins, including ZO-1, occludin, and claudins. Microbial protein and amino acid metabolism influences nitrogen utilization and the production of ammonia and other metabolites, with implications for nutrient efficiency and environmental emissions. Microbial transformation of bile acids generates metabolites that regulate host metabolism through FXR and TGR5 signaling. In parallel, microbial-associated molecular patterns can modulate immune responses through Toll-like receptor and NF-κB-related pathways and influence inflammatory mediators, including IL-1β, IL-6, and TNF-α. These interconnected mechanisms ultimately affect nutrient absorption, energy utilization, intestinal integrity, immune homeostasis, feed efficiency, growth performance, carcass traits, meat quality, and resilience to environmental stress.

The biological significance of the poultry gut microbiota extends beyond microbial community composition to its functional interactions with dietary substrates and host physiology. Through the fermentation of otherwise indigestible nutrients, transformation of amino acids and bile acids, production of bioactive metabolites, and modulation of epithelial and immune signaling, the gut microbiota can influence nutrient availability, intestinal homeostasis, metabolic efficiency, and production performance (Pan, et al., 2014). However, these effects are highly context-dependent and may vary according to poultry species, intestinal segment, developmental stage, diet, environmental conditions, and host genetic background. Therefore, the relationship between gut microbiota and production phenotypes should be interpreted as a complex diet–microbiota–host interaction rather than as a direct consequence of changes in individual microbial taxa (Fig. 2).

### Energy and carbohydrate metabolism

Dietary carbohydrates that escape digestion in the upper gastrointestinal tract provide substrates for microbial fermentation, particularly in the ceca. Microbial degradation of complex carbohydrates and NSP generates SCFAs, including acetate, propionate, and butyrate, which can contribute to energy recovery and intestinal epithelial metabolism (Jha, et al., 2021). Butyrate is an important energy substrate for intestinal epithelial cells and may support mucosal integrity, whereas acetate and propionate can participate in systemic energy metabolism and signaling pathways (Borda-Molina, et al., 2018).

In addition to serving as metabolic substrates, SCFAs function as signaling molecules through GPR41 (FFAR3) and GPR43 (FFAR2), influencing epithelial metabolism, immune responses, and inflammatory processes. SCFAs may also affect intestinal motility and enteroendocrine signaling, thereby indirectly influencing nutrient utilization (Pan, et al., 2014) (Borda-Molina, et al., 2018). These mechanisms provide a potential link between microbial carbohydrate fermentation and production traits such as feed efficiency and growth performance.

However, increased SCFA production should not automatically be interpreted as evidence of improved host energy utilization. The physiological consequences of microbial fermentation depend on the amount and type of substrate fermented, the intestinal location of fermentation, the microbial pathways involved, and the host's capacity to utilize the resulting metabolites. Thus, future studies should evaluate microbial fermentation together with metabolite flux, host energy balance, and production outcomes rather than relying solely on microbial abundance or predicted metabolic pathways.

### Protein and amino acid utilization

Microbial metabolism of dietary protein and amino acids represents an important interface between poultry nutrition, intestinal health, and environmental sustainability. Protein that escapes digestion in the small intestine can be metabolized by intestinal microorganisms, generating ammonia, BCFAs, phenols, indoles, amines, and other metabolites. Although some microbial amino acid metabolites may exert physiological effects, excessive proteolytic fermentation may contribute to intestinal dysfunction and increase nitrogen losses (Apajalahti J, 2004; Borda-Molina, et al., 2018).

Precision protein nutrition may therefore influence both microbial metabolism and host nitrogen utilization. Reducing dietary crude protein while maintaining adequate digestible essential amino acids can decrease the amount of protein available for excessive microbial fermentation in the distal intestine. This may shift microbial metabolism toward carbohydrate fermentation and reduce the production of ammonia and other potentially deleterious metabolites.

The effects of protein reduction, however, depend on the degree of crude protein reduction, amino acid balance, protein digestibility, and availability of fermentable carbohydrates. Excessive protein restriction or inadequate supplementation of limiting amino acids may compromise growth and production performance. Therefore, the relationship between dietary protein and microbial nitrogen metabolism should be evaluated using integrated measures of amino acid digestibility, microbial functional pathways, nitrogen retention, and production efficiency.

At the farm level, improved nitrogen utilization may also reduce nitrogen excretion and ammonia emissions. Nevertheless, the contribution of intestinal microbial metabolism to total ammonia emissions is influenced by subsequent manure microbial processes, housing conditions, litter management, and ventilation. Therefore, microbial nitrogen metabolism should be considered one component of a broader nitrogen-emission pathway rather than the sole determinant of farm-level ammonia production.

### Lipid metabolism and bile acid transformation

Gut microorganisms participate in the transformation of bile acids and may thereby influence host lipid metabolism and energy homeostasis (Pan, et al., 2014). Primary bile acids synthesized by the host can be modified by intestinal microorganisms into secondary bile acids with distinct biological activities. These microbial transformations may affect host signaling through receptors such as the farnesoid X receptor (FXR) and Takeda G protein-coupled receptor 5 (TGR5), which regulate bile acid homeostasis, lipid metabolism, glucose metabolism, and energy expenditure (Wahlstrom, et al., 2016).

Dietary lipid composition may further influence these interactions by altering bile acid secretion and the availability of substrates for microbial metabolism. Polyunsaturated fatty acids, particularly n-3 PUFA, may modulate microbial community structure and inflammatory signaling and may contribute to intestinal barrier maintenance (Sijben, et al., 2000). These interactions provide a potential mechanistic link between dietary lipid nutrition, microbial metabolism, and host energy regulation.

Nevertheless, the relationship between gut microbiota and lipid metabolism in poultry remains less well characterized than microbial carbohydrate fermentation. Future studies should integrate microbial metagenomics, bile acid profiling, lipidomics, and host metabolic phenotyping to determine how specific microbial pathways influence lipid utilization and whether these effects translate into measurable improvements in growth, body composition, or meat quality.

### Intestinal barrier integrity and nutrient absorption

The intestinal epithelium forms a critical interface between the host and the microbial ecosystem. Gut microbiota and their metabolites can influence epithelial cell proliferation, mucus production, tight-junction organization, and intestinal permeability (Pan, et al., 2014). SCFAs, particularly butyrate, may support epithelial energy metabolism and regulate the expression and assembly of tight-junction proteins, including ZO-1, occludin, and claudins (Peng, et al., 2009).

Maintenance of intestinal barrier integrity is essential for efficient nutrient absorption and prevention of excessive translocation of microbial products. Dysbiosis, epithelial damage, or increased intestinal permeability may increase exposure to microbial-associated molecular patterns and promote inflammatory responses, thereby diverting nutrients and metabolic energy away from growth and production (Awad, et al., 2017; Kogut, et al., 2018).

The effects of the microbiota on nutrient absorption may also involve regulation of nutrient transporter expression and activity. Microbial metabolites and host signaling pathways may influence the absorption of glucose, amino acids, and lipids, although the extent and specificity of these effects in poultry remain incompletely characterized. Thus, improved production performance associated with microbiota modulation may result not only from enhanced microbial degradation of dietary substrates but also from improved host capacity to absorb and utilize nutrients.

### Immune regulation and disease resistance

The gut microbiota plays an important role in maintaining intestinal immune homeostasis. Microbial-associated molecular patterns interact with host pattern-recognition receptors and can influence signaling pathways involving Toll-like receptors, NF-κB, and inflammatory cytokines such as IL-1β, IL-6, and TNF-α (Kogut, et al., 2018). Appropriate microbial stimulation is essential for immune maturation and pathogen resistance, whereas excessive inflammatory activation may impair intestinal function and production efficiency (Kogut, et al., 2016).

A balanced microbiota may contribute to pathogen resistance through competitive exclusion, production of antimicrobial metabolites, modulation of mucosal immunity, and maintenance of epithelial barrier integrity. Conversely, dysbiosis may increase susceptibility to enteric pathogens and promote chronic low-grade inflammation.

The production consequences of microbiota-mediated immune regulation may be particularly important in rapidly growing broilers. Persistent inflammation can increase metabolic demands and divert nutrients from tissue deposition toward immune responses, potentially reducing growth and feed efficiency (Kogut, et al., 2018). Therefore, microbiota regulation that supports immune homeostasis may improve production performance indirectly by reducing the metabolic costs associated with chronic inflammation.

However, microbial diversity should not be considered synonymous with immune health. A more diverse microbial community is not necessarily more protective, and microbial effects on immunity depend on community function, metabolite production, pathogen exposure, and host immune status. Functional validation is therefore required before specific microbial taxa can be considered reliable biomarkers of disease resistance or production efficiency.

### Implications for poultry production performance

#### Feed efficiency

Feed efficiency is one of the most economically important traits in poultry production and is commonly evaluated using FCR and related measures of feed intake relative to growth or egg production (Zuidhof, et al., 2014). The relationship between gut microbiota and feed efficiency is likely mediated by multiple interacting mechanisms rather than by a single microbial taxon.

Potential mechanisms include improved degradation of complex dietary carbohydrates, enhanced SCFA production and energy recovery, reduced excessive protein fermentation, improved intestinal barrier integrity, enhanced nutrient absorption, modulation of nutrient transporter activity, reduced chronic inflammation, and improved bile acid metabolism. Collectively, these processes may increase the proportion of dietary nutrients that are available for productive functions (Jha, et al., 2021; Pan, et al., 2014).

However, associations between microbial composition and FCR should not be interpreted as evidence of causality. A microbial signature associated with improved FCR may reflect differences in feed intake, growth rate, dietary composition, host genotype, or environmental exposure rather than directly causing improved feed efficiency. Therefore, identifying functional microbial biomarkers that predict feed efficiency across independent populations will require longitudinal studies, controlled interventions, and validation under commercial conditions.

From an economic perspective, improvements in feed efficiency may reduce feed requirements and feed costs per unit of poultry product. However, the economic value of microbiota-targeted interventions should be evaluated using both biological and financial outcomes, including feed cost, body weight gain, egg mass, mortality, medication use, and the cost of the intervention itself.

#### Growth performance

Growth performance is determined by the integrated effects of nutrient digestibility, energy utilization, intestinal health, immune status, and metabolic efficiency. Gut microbiota can influence each of these processes and may therefore contribute to variation in body weight gain and FCR.

In broilers, microbial fermentation of otherwise inaccessible dietary substrates may increase energy recovery, whereas maintenance of epithelial barrier integrity may improve nutrient absorption and reduce nutrient losses associated with intestinal dysfunction (Jha, et al., 2021). Microbiota-mediated immune regulation may further reduce the metabolic costs of chronic inflammation (Kogut, et al., 2016). Together, these mechanisms provide a biological basis for associations between gut microbial function and growth performance.

Nevertheless, growth-promoting effects are not universal across poultry species. Broilers, layers, ducks, geese, and turkeys differ substantially in growth trajectory, nutrient requirements, digestive physiology, and production objectives. A microbial feature associated with rapid growth in broilers may therefore have limited relevance to laying hens or waterfowl. Species-specific and stage-specific validation is essential before microbiota-based nutritional interventions are translated across poultry production systems.

#### Carcass traits and meat quality

The potential effects of gut microbiota extend beyond live performance to carcass composition and product quality. Improved nutrient utilization and energy partitioning may influence protein deposition, adipose tissue development, and body composition, thereby affecting carcass yield and tissue distribution (P. Celi, 2017).

Microbial metabolites may also influence systemic inflammatory and oxidative pathways that indirectly affect skeletal muscle metabolism (P. Celi, 2017). Changes in microbial activity may therefore have potential consequences for meat quality traits, including intramuscular fat, tenderness, water-holding capacity, and oxidative stability.

However, direct evidence linking specific gut microbial functions to carcass and meat quality remains limited compared with evidence for growth performance and intestinal health. Future studies should integrate gut microbiome profiling with carcass composition, muscle metabolomics, oxidative stress markers, and standardized meat quality measurements. Such approaches may clarify whether microbiota-targeted nutritional interventions can consistently improve both production efficiency and the quality of poultry products.

#### Heat stress adaptation

Heat stress is a major challenge to intensive poultry production and is expected to become increasingly important under global climate change. Elevated environmental temperature can reduce feed intake, alter intestinal blood flow, impair epithelial barrier integrity, increase oxidative stress, and disrupt immune homeostasis (Lara, et al., 2013).

Heat stress may also induce microbial dysbiosis and increase intestinal permeability, thereby facilitating the translocation of microbial products and amplifying inflammatory responses (Sohail, et al., 2010). This interaction can create a feedback loop in which environmental stress promotes intestinal dysfunction, while impaired intestinal health further compromises nutrient utilization and production performance.

Microbiota-targeted strategies, including probiotics, prebiotics, synbiotics, dietary fiber optimization, and other nutritional interventions, may improve heat-stress resilience by supporting epithelial integrity, modulating inflammation, and promoting beneficial microbial metabolites. However, intervention efficacy may vary with environmental temperature, humidity, bird age, genotype, diet, and baseline microbiota.

Therefore, microbiota-based strategies for heat-stress mitigation should be evaluated under realistic environmental and commercial production conditions. Studies should simultaneously assess microbial changes, intestinal permeability, oxidative stress, immune responses, feed intake, FCR, growth, and mortality to determine whether microbial modulation produces meaningful improvements in heat-stress resilience.

#### Nitrogen utilization and ammonia emission

Poultry production generates substantial nitrogen-containing waste, and microbial metabolism contributes to the transformation of nitrogenous compounds throughout the gastrointestinal tract and manure environment. Excess dietary protein that escapes host digestion may undergo microbial fermentation, generating ammonia and other nitrogen-containing metabolites (Apajalahti J, 2004; Nahm, 2007).

Improving dietary protein and amino acid utilization may therefore reduce the amount of nitrogen available for excessive microbial fermentation and subsequent nitrogen losses. Precision protein nutrition, enhanced amino acid digestibility, exogenous enzyme supplementation, and targeted modulation of microbial fermentation may collectively improve nitrogen efficiency while maintaining production performance.

However, the relationship between intestinal microbiota and farm-level ammonia emissions is complex. Ammonia generation continues after excretion through microbial degradation of nitrogenous compounds in manure and litter and is strongly influenced by moisture, temperature, pH, ventilation, and housing systems. Therefore, the quantitative contribution of intestinal microbial metabolism to total ammonia emissions remains insufficiently defined.

Future studies should integrate dietary nitrogen balance, microbial metabolomics, manure microbiology, litter characteristics, housing conditions, ventilation, and direct ammonia measurements. Such integrated approaches are necessary to determine whether microbiota-informed nutritional strategies can generate measurable reductions in farm-level ammonia emissions.

#### Sustainable and antibiotic-free poultry production

Gut health is increasingly recognized as an important component of poultry welfare. Intestinal dysfunction may increase susceptibility to disease, impair nutrient utilization, increase physiological stress, and reduce resilience to environmental challenges (Kogut, et al., 2016). Microbiota-targeted interventions that maintain epithelial barrier integrity and immune homeostasis may therefore contribute indirectly to improved welfare by reducing disease burden and physiological stress.

However, welfare outcomes should not be inferred solely from microbial composition or production performance. Comprehensive assessment should include behavioral indicators, morbidity, mortality, physiological stress markers, disease incidence, environmental conditions, and other validated welfare measures.

Microbiota regulation may also support antibiotic-free or reduced-antibiotic production systems. Probiotics, prebiotics, synbiotics, postbiotics, and other microbiota-targeted strategies may help maintain intestinal health and resistance to enteric pathogens, potentially reducing the need for routine antimicrobial use. Nevertheless, microbiota-based interventions should be viewed as components of integrated health-management programs rather than direct replacements for antimicrobial therapy under all circumstances.

The practical success of antibiotic-free production will depend on combining microbiota-targeted nutrition with biosecurity, vaccination, environmental management, pathogen control, and appropriate husbandry. The effectiveness of such systems should therefore be evaluated using comprehensive indicators that include production performance, disease incidence, antimicrobial use, animal welfare, and economic sustainability.

## Nutritional strategies targeting poultry gut microbiota

With increasing restrictions on antibiotic growth promoters, nutritional modulation of the gut microbiota has become an important strategy for improving intestinal health and production efficiency in poultry (Kogut, 2019). Unlike pharmaceutical interventions, nutritional approaches can be integrated into routine feed formulation and management, making them potentially more practical for large-scale production. These strategies aim not simply to increase the abundance of individual beneficial taxa but to shape microbial functions, metabolic pathways, and host–microbiota interactions that contribute to nutrient utilization, intestinal homeostasis, and production performance. However, their efficacy is often dependent on poultry species, age, basal diet, environmental conditions, intervention dose, and the resident microbiota. Therefore, precision microbiota regulation requires strategies tailored to specific production stages and physiological contexts (Table 3).

**Table 3:** Representative nutritional and microbiota-targeted interventions in poultry.

Intervention	Poultry species	Inclusion level/dose	Duration	Microbial alterations	Functional pathways/metabolites	Production outcomes	Key references
Probiotics (Lactobacillus plantarum B1)	Broilers (Ross 308)	1 × 10^9^ CFU/kg feed	1–42 days of age	Reduced abundance of potentially pathogenic bacteria (e.g., *Escherichia coli*); increased lactic acid bacteria counts	Improved intestinal microbial balance; Improved intestinal microbial balance; reduced potentially pathogenic bacteria	Improved ADG and feed efficiency; finisher-period or full-period feeding most effective	(Peng, et al., 2016)
Probiotics (multi-strain DFM)	Broilers/Layers	Variable (strain-dependent)	Variable	Modification of intestinal microbiota composition; increased beneficial bacteria	SCFA production; immune stimulation; reduction of inflammatory reactions; prevention of pathogen colonization	Enhanced nutrient utilization; improved growth and laying performance; gut health improvement	(Jha, et al., 2020)
Prebiotics (refined functional carbohydrate + yeast culture)	Broilers	As fed (commercial dose)	1–42 days of age	Reduced cecal Campylobacter colonization	Reduced cecal pathogen colonization	Improved ADG	(Froebel, et al., 2019)
Prebiotics (oligosaccharides as fiber supplement)	Broilers	Variable (isolated oligosaccharide form)	Variable	Enhanced beneficial carbohydrate-utilizing bacteria (e.g., *Lactobacillus* and *Bifidobacterium*)	Modulation of gut immunity; increased SCFA production in the gut	Improved growth performance and feed efficiency; potential alternative to antibiotic growth promoters	(Singh, et al., 2021); (Pourabedin, et al., 2015)
Synbiotics (Bacillus subtilis + B. licheniformis + C. butyricum + yeast cell wall + XOS)	Broilers (Arbor Acres Plus)	1.5 g/kg diet	1–42 days of age	Not specified in available data	Enhanced antioxidant capacity and immune homeostasis	Improved average daily gain and feed efficiency; increased breast yield; decreased abdominal fat	(Cheng, et al., 2017)
Synbiotics (Bacillus subtilis + XOS + MOS)	Broilers (Cobb 48)	Probiotics 1 g/kg + XOS 150 mg/kg + MOS 1 g/kg	1–42 days of age	Not specified in available data	Enhanced intestinal barrier function; improved mucosal immune response and antioxidant capacity	Improved growth performance and feed efficiency; increased villus height-to-crypt depth ratio in duodenum, jejunum and ileum	(Min, et al., 2016)
Resistant starch (potato starch)	Meat ducks	12% dietary inclusion	Growing period	Altered gut microbiome composition	Increased cecal acetate, propionate, and butyrate; improved ileal barrier function	Reduced plasma IL-1β and IL-6; improved intestinal health	(Qin, et al., 2019)
Low-protein diet + protease	Broilers (Hubbard)	positive control 19.3% crude protein vs. negative control 18.8% CP; + 0.05% protease	1–42 days of age	Not directly measured	Improved nitrogen retention; enhanced protein digestion	Protease supplementation partially restored growth performance under low-protein diets; improved feed efficiency and nitrogen retention	(Yameen, 2017)
Low-protein diet + protease	Broilers	normal-protein vs. low-protein (1% CP reduction + ∼8–12% amino acid reduction); ± exogenous protease 1 g/kg	8–35 days of age	Not directly measured	Improved protein digestibility and nutrient utilization	Exogenous protease supplementation improved growth performance under low-protein diets	(Ndazigaruye, et al., 2019)
Amino acid optimization (Met, Lys, Ile, Thr deficiency)	Laying hens (Peking Pink)	Met 0.25% vs. 0.46%; Lys 0.56% vs. 0.76%; Ile 0.54% vs. 0.72%; Thr 0.46% vs. 0.56%	38–50 weeks of age (12 weeks)	Altered gut microbiota composition associated with AA deficiency	Amino acid metabolism; gut microbiome–performance correlations	Reduced laying performance and egg quality with AA deficiency	(Geng, et al., 2021)
Plant-derived alkaloids (Macleaya cordata extract, MCE) as antibiotic alternative	Broilers	As growth promoter (dose not specified in paper)	Not specified	Altered intestinal microbiota composition; modulation of foregut and hindgut microbiota	Growth promotion through modulation of intestinal microbiota composition and metabolic potential	Potential alternative to antibiotic growth promoters	(Huang, et al., 2018)

### Probiotics, prebiotics and synbiotics

Probiotics are live microorganisms that confer health benefits when administered in adequate amounts. Commonly investigated probiotic organisms in poultry include strains of *Lactobacillus, Bifidobacterium, Bacillus, Enterococcus*, and *yeast* (Mountzouris, et al., 2007). Their potential benefits include competitive exclusion of pathogens, production of antimicrobial compounds, modulation of intestinal barrier function, and regulation of immune responses and microbial metabolite production. Some probiotic strains may also enhance SCFA production and improve nutrient utilization, thereby contributing to growth performance and feed efficiency (Gadde, et al., 2017).

However, probiotic effects are highly strain-specific and should not be generalized at the genus level. Efficacy may depend on strain identity, viability, dose, delivery matrix, storage stability, host age, basal diet, and the composition of the resident microbiota. Consequently, a probiotic that improves performance under experimental conditions may not produce equivalent effects across commercial farms. Future studies should therefore prioritize strain-level characterization and functional validation rather than relying solely on taxonomic classification.

Prebiotics are selectively utilized substrates that promote the growth or activity of beneficial microorganisms. Common examples include fructooligosaccharides, galactooligosaccharides, and xylooligosaccharides, which can support microbial fermentation and SCFA production and may influence intestinal morphology, nutrient absorption, and immune function (Huyghebaert, et al., 2011). However, prebiotic effects depend on substrate structure, dose, intestinal accessibility, and the metabolic capacity of the resident microbiota. Therefore, the same prebiotic may produce different outcomes among poultry species or production stages.

Synbiotics combine probiotics and prebiotics with the aim of generating complementary or synergistic effects. By providing both a beneficial microbial strain and a suitable substrate, synbiotics may improve microbial establishment, enhance SCFA production, support intestinal barrier function, and improve nutrient utilization and production efficiency (Awad, et al., 2009). Evidence from poultry studies suggests that appropriately selected probiotic–prebiotic combinations can improve microbial balance, intestinal integrity, immune regulation, and growth-related outcomes (Shang, et al., 2018). However, successful synbiotic effects remain highly dependent on compatibility between the probiotic strain and prebiotic substrate. Differences in microbial substrate utilization, dietary composition, host species, age, and experimental conditions may account for inconsistent responses among studies. For example, synbiotic combinations involving *Lactobacillus* strains with fermentable oligosaccharides, such as fructooligosaccharides (FOS) or mannan-oligosaccharides (MOS), have been investigated in poultry and shown potential benefits in promoting beneficial microbial colonization, enhancing SCFA production, improving intestinal barrier function, and supporting growth performance (Awad, et al., 2009). Similarly, *Bacillus*-based probiotics combined with prebiotic substrates have been reported to improve microbial stability and intestinal health under specific dietary and management conditions. However, the reproducibility of these effects remains dependent on strain characteristics, prebiotic utilization capacity, supplementation level, and farm-specific environmental factors (Gadde, et al., 2017). Thus, systematic evaluation of strain–substrate compatibility and validation under commercial production conditions are required before synbiotics can be broadly applied as standardized microbiota-targeted interventions.

### Dietary fiber and exogenous enzymes

Dietary fiber manipulation provides a practical approach to regulating microbial fermentation by modifying the availability and physicochemical characteristics of microbial substrates. Selective inclusion of fermentable fiber may promote carbohydrate-degrading microorganisms and increase the production of SCFAs, particularly butyrate, which can support epithelial energy metabolism and intestinal barrier integrity. However, the effects of dietary fiber depend strongly on fiber source, solubility, viscosity, particle size, inclusion level, and bird age (Jha, et al., 2021). Different dietary fiber fractions may therefore exert distinct regulatory effects on microbial ecology. Soluble non-starch polysaccharides (NSPs), including β-glucans and arabinoxylans, can provide fermentable substrates for microbial carbohydrate metabolism and promote SCFA-producing bacteria, whereas insoluble fibers such as cellulose mainly influence intestinal motility and microbial habitat structure (Knudsen, 2014). Through modulation of microbial fermentation patterns, dietary fibers may alter SCFA profiles, epithelial energy metabolism, and intestinal barrier function. Therefore, optimization of fiber composition and inclusion level should consider both microbial responses and physiological requirements at different production stages.

Exogenous enzymes, including xylanase and β-glucanase, may enhance the accessibility of otherwise poorly digestible substrates and thereby alter the substrate supply available to intestinal microorganisms. By reducing digesta viscosity and increasing the release of fermentable carbohydrates, enzyme supplementation may promote beneficial microbial fermentation and improve nutrient utilization (M.R. Bedford, 2012). Nevertheless, enzyme effects are dependent on the composition of the basal diet and the physicochemical characteristics of dietary fiber. Therefore, fiber–enzyme combinations should be optimized according to diet composition and production stage rather than applied uniformly across poultry production systems.

Age-specific fiber management may provide additional opportunities for precision microbiota regulation. During the grower stage, targeted fiber supplementation may support microbial fermentation and intestinal development while improving nutrient utilization. In laying hens, dietary strategies that maintain microbial stability and intestinal function may contribute to sustained nutrient absorption and long-term egg production (Jha, et al., 2021). These examples highlight the importance of aligning dietary fiber interventions with the physiological and production objectives of each stage of the poultry production cycle.

### Precision protein nutrition

Precision protein nutrition represents an important strategy for simultaneously regulating gut microbial metabolism, nutrient utilization, and environmental sustainability. Reducing dietary crude protein while maintaining adequate levels of essential amino acids can decrease the amount of undigested protein reaching the distal intestine and thereby reduce excessive proteolytic fermentation and ammonia production (Bregendahl, et al., 2002). In practical poultry nutrition, moderate crude protein reduction strategies are generally accompanied by supplementation of limiting amino acids, including lysine, methionine, threonine, and tryptophan, to maintain growth performance while decreasing nitrogen availability for microbial protein fermentation (Kumar, et al., 2016). Reduced availability of undigested protein substrates may decrease the abundance or activity of proteolytic microbial populations and shift microbial metabolism toward carbohydrate fermentation, thereby increasing beneficial SCFA production and reducing accumulation of ammonia and other nitrogenous metabolites (Namroud, et al., 2008).

However, the benefits of reduced-protein diets depend on the degree of protein reduction and the accuracy of amino acid balancing. Excessive protein restriction or inadequate supplementation of limiting amino acids may compromise growth, feed efficiency, immune function, or production performance. Therefore, precision protein nutrition should focus on matching digestible amino acid supply to the physiological requirements of birds rather than simply minimizing crude protein concentration.

From a microbiota perspective, reduced protein availability in the distal intestine may shift microbial metabolism from proteolytic toward carbohydrate fermentation, potentially increasing SCFA production while reducing the accumulation of ammonia and other potentially harmful protein-fermentation products. The specific responses may also depend on the balance among lysine, methionine, threonine, tryptophan, and other amino acids, as well as the availability of fermentable carbohydrates (Apajalahti J, 2004; Hilliar, et al., 2020). Thus, future studies should integrate dietary nitrogen balance with microbial metagenomics and metabolomics to determine how specific protein and amino acid formulations alter microbial metabolic pathways.

This strategy may also contribute to environmental sustainability by reducing unnecessary nitrogen excretion. However, the extent to which changes in intestinal microbial nitrogen metabolism translate into reductions in farm-level ammonia emissions remains insufficiently defined. Commercial-scale studies integrating dietary nitrogen utilization, manure microbiology, housing ventilation, and direct ammonia measurements are therefore needed to establish this relationship.

### Lipids and micronutrients

Dietary lipid manipulation represents another potential approach to microbiota regulation. The type and amount of dietary fatty acids can influence microbial membrane metabolism, bile acid transformation, inflammatory signaling, and host energy metabolism. Polyunsaturated fatty acids, particularly n-3 PUFA, may modify microbial community structure and inflammatory responses and may contribute to intestinal barrier maintenance (Sijben, et al., 2000). However, the effects of specific lipid sources remain variable and may depend on fatty acid composition, inclusion level, bird age, and basal diet.

Micronutrients, including zinc, selenium, and vitamins A and E, may also influence host–microbiota interactions through effects on antioxidant capacity, immune function, and intestinal barrier integrity (P.F. Surai, 2014). Nevertheless, microbiota responses to micronutrient supplementation are highly dependent on dose, chemical form, baseline nutritional status, and interactions with other dietary components.

Therefore, lipid and micronutrient interventions should be considered as components of integrated nutritional programs rather than isolated microbiota-targeted treatments.

### Emerging microbiota-targeted approaches

Beyond conventional nutritional interventions, emerging approaches offer opportunities for more direct manipulation of poultry gut microbial ecosystems. Fecal microbiota transplantation (FMT) aims to introduce complex microbial communities from selected donors, whereas bacteriophage-based strategies can selectively target specific bacterial populations while preserving broader microbial diversity (Ducarmon, et al., 2019; Lin, et al., 2017). In addition, IgA-mediated host–microbiota interactions provide an endogenous mechanism through which the host immune system can shape microbial colonization and maintain intestinal ecosystem stability (Corthesy, 2013; Mantis, et al., 2011).

Although these approaches have potential for precision microbiota regulation, their application in commercial poultry production remains limited. FMT requires stringent donor selection, pathogen screening, and standardized procedures to ensure microbial stability and biosafety. Variations in donor microbiota composition, host status, diet, and environmental conditions may affect the reproducibility of outcomes. Bacteriophage-based interventions face challenges including host specificity, bacterial resistance, phage stability, and efficient intestinal delivery. Similarly, IgA-based microbiota modulation requires a deeper understanding of immune–microbiota interactions and long-term ecological effects. Therefore, these approaches should currently be considered emerging technologies rather than established production strategies.

Successful translation of these microbiota-targeted interventions will require further evaluation of scalability, manufacturing consistency, economic feasibility, and regulatory requirements. Future studies should integrate multi-omics analyses, controlled intervention trials, and commercial-scale validation to determine whether these strategies can provide reliable and cost-effective benefits for sustainable poultry production.

Nutritional regulation of the gut microbiota should be viewed as a bidirectional process rather than a one-way effect of diet on microbial composition. Dietary substrates determine which microorganisms and metabolic pathways are favored, whereas microbial communities, in turn, influence the digestion, metabolism, and utilization of nutrients by the host. Microbial degradation of complex carbohydrates expands the host's capacity to access otherwise unavailable substrates, while microbial metabolism of amino acids and lipids generates metabolites that can influence host energy metabolism and intestinal physiology.

Microbial-derived metabolites represent important mediators of these reciprocal interactions. SCFAs, branched-chain fatty acids (BCFAs), bile acid derivatives, and other microbial products can influence epithelial metabolism, intestinal barrier function, immune regulation, and systemic energy homeostasis (Borda-Molina, et al., 2018). Thus, the consequences of a dietary intervention cannot be fully predicted from changes in microbial abundance alone. Instead, nutritional effects should be evaluated by integrating microbial composition, metabolic activity, metabolite production, and host physiological responses.

This bidirectional perspective has important implications for precision poultry nutrition. Rather than attempting to establish a universal "optimal" microbial community, future interventions should aim to promote functional microbial activities that complement host nutrient metabolism and support production objectives. Such an approach may enable dietary strategies to be tailored according to poultry species, developmental stage, genetic background, basal diet, and environmental conditions.

## Current limitations and future perspectives

Despite rapid advances in poultry microbiome research, substantial challenges remain in translating microbial associations into reliable biological mechanisms and practical production strategies. These challenges arise from limitations in study design, sequencing technologies, functional validation, experimental reproducibility, and commercial implementation. Addressing these issues will require a shift from descriptive characterization of microbial communities toward causal, functional, and precision-based approaches that integrate microbiology with poultry nutrition, host physiology, and production management (Table 4).

**Table 4:** Translational potential, limitations, and sustainability considerations of microbiota-targeted strategies in poultry.

Strategy	Primary application	Potential benefits	Major limitations	Biosafety/regulatory considerations	Scalability	Sustainability relevance	Key references
Precision nutrition	Feed efficiency optimization; species-specific nutrient utilization	Improved nutrient utilization and feed efficiency; potential reduction of nutrient losses	Requires species-, age-, and production stage-specific formulation; microbiota response variability between flocks	Low (conventional feed ingredients)	High (integrated into existing feed systems)	Reduced feed waste and environmental nutrient pollution	(Yadav, et al., 2019); (Kogut, 2019)
Probiotics	Gut health maintenance; antibiotic-free production; pathogen exclusion	Enhanced intestinal barrier function; immune stimulation; competitive exclusion of pathogens; SCFA production	Strain-specific efficacy; variable colonization capacity; storage stability requirements; host-specificity of optimal strains	Moderate (GRAS/QPS status for well-characterized strains; novel strains require safety evaluation and regulatory assessment)	High (commercial products available; integration into feed or water)	Reduced antimicrobial dependence; supports antibiotic-free production systems	(Yadav, et al., 2019); (Fathima, et al., 2022); (Khan, et al., 2020); (Johnson, et al., 2023)
Prebiotics	Selective stimulation of beneficial microbiota; microbial functional modulation	Enhanced SCFA production; selective stimulation of beneficial carbohydrate-utilizing microbiota; improved gut immune modulation	Efficacy dependent on resident microbiota composition; dose-response variability; limited direct pathogen inhibition	Low (food-grade carbohydrates)	High (easily incorporated into feed formulations)	Improved nutrient utilization; potential reduction of antibiotic use	(Yadav, et al., 2019); (Fathima, et al., 2022)
Synbiotics	Combined probiotic–prebiotic microbial ecosystem modulation	Potential synergistic enhancement of probiotic survival and activity; improved antioxidant and immune responses	Compatibility between probiotic strains and prebiotic substrates; reproducibility across production conditions; limited mechanistic understanding	Moderate (requires safety assessment of combined formulation)	Moderate to high (commercial formulations available, but efficacy depends on strain–substrate compatibility)	Improved production efficiency; reduced need for therapeutic interventions	(Fathima, et al., 2022); (Yadav, et al., 2019)
Postbiotics	Microbial metabolite-based gut health intervention	Greater stability and standardization than live organisms; defined composition; no colonization requirement	Evolving product definition; limited poultry-specific efficacy data; mechanism-of-action characterization incomplete	Regulatory classification varies depending on product composition and jurisdiction	High potential (heat-stable; long shelf life; no cold chain)	Potential for resource-efficient production; reduced biological variability	(Salminen, et al., 2021)
Fecal/cecal microbiota transplantation (FMT/CMT; experimental approach)	Early-life microbial programming; pathogen colonization resistance	Transfer of complex microbial communities; potential improvement of microbial maturation and pathogen resistance	Donor variability; risk of pathogen and AMR gene transmission; lack of standardized protocols; batch-to-batch inconsistency	High biosafety concern (potential transmission of MDR organisms, virulence factors); regulatory frameworks largely undefined for livestock applications	Low (labor-intensive; requires donor screening; difficult to standardize for large flocks)	Currently limited; potential contribution to antibiotic reduction if biosafety and standardization challenges are overcome	(Pottenger, et al., 2023); (Clavijo, et al., 2022)
Bacteriophages	Pathogen-specific biocontrol (*Salmonella*, Campylobacter, *E. coli*)	High host specificity; minimal disruption of commensal microbiota; potentially effective at farm and processing levels	Narrow host range; bacterial resistance development; phage stability in GI environment; cocktail design complexity	Regulatory approval required (variable by jurisdiction); some products approved as processing aids (e.g., GRAS in the USA); therapeutic use less established	Moderate (encapsulation and feed-delivery technologies under development; some commercial products available)	Reduced broad-spectrum antimicrobial use; targeted intervention minimizes ecological disruption	(Abd-El Wahab, et al., 2023); (Zbikowska, et al., 2020); (Lorenzo-Rebenaque, et al., 2022); (Clavijo, et al., 2019)
IgA–microbiota interaction-based regulation	Precision mucosal immune–microbiota interface modulation	Potential regulation of microbial community structure through IgA-mediated host selection; maintenance of mucosal homeostasis	Production and delivery of functional IgA at scale; stability in GI environment; limited in vivo poultry data; mechanism primarily characterized in mammalian models	Biologics regulation applicable; would require extensive safety and efficacy evaluation	Currently low (proof-of-concept stage; no commercial poultry products)	Potential for highly precise, low-residue microbiota management if translational barriers overcome	(Pabst, 2012); (Palm, et al., 2014); (Bunker, et al., 2015); (Moor, et al., 2017)

### From association to causality

A major limitation of current poultry microbiome research is the predominance of observational studies. Numerous studies have identified associations between specific microbial taxa and growth performance, feed efficiency, disease resistance, or other production traits. However, such associations do not establish causality. A microbial taxon enriched in high-performing birds may directly contribute to improved nutrient metabolism, or it may simply reflect differences in diet, feed intake, growth rate, immune status, or intestinal environment.

Establishing causality therefore requires experimental approaches that move beyond correlation-based microbiome profiling. Controlled microbial interventions, longitudinal sampling, targeted manipulation of microbial metabolites, defined microbial consortia, and functional validation should be combined to determine whether specific microorganisms or microbial pathways directly influence host phenotypes. Transplantation experiments may further test whether microbial communities or their metabolites can reproducibly transfer specific physiological or production traits.

However, causal inference in poultry remains challenging because complex microbial communities interact with multiple host and environmental factors. Gnotobiotic and defined-community models may provide mechanistic insights but do not fully reproduce the complexity of commercial poultry production. Therefore, findings obtained from reductionist models should ultimately be validated in conventional birds under controlled and commercial conditions.

### Limitations of sequencing approaches

16S rRNA gene sequencing remains widely used because of its relatively low cost, high throughput, and broad applicability. However, its taxonomic resolution is often insufficient for distinguishing closely related species or strains with substantially different functional capacities. Primer selection, amplification bias, sequencing depth, and bioinformatic pipelines may further influence the observed community structure (Oakley, et al., 2014b). Differences in 16S rRNA variable regions and analytical methods can therefore contribute to apparently inconsistent findings among studies.

Shotgun metagenomic sequencing provides broader information on microbial genomes, functional genes, and taxonomic composition and can offer greater resolution than 16S rRNA profiling. Nevertheless, metagenomics is more expensive and computationally demanding and does not necessarily indicate whether detected genes are actively expressed or whether the corresponding metabolites are produced in vivo (Qu, et al., 2008). Functional potential should therefore not be equated with functional activity.

The two approaches should consequently be viewed as complementary rather than interchangeable. 16S rRNA sequencing may be appropriate for large-scale community profiling and hypothesis generation, whereas shotgun metagenomics is more suitable for investigating microbial functional potential. Neither approach alone, however, is sufficient to establish active microbial metabolism or causal relationships with host phenotypes.

### Multi-omics integration and functional validation

A major knowledge gap in poultry microbiome research is the limited connection between microbial composition, microbial activity, and host physiology. Taxonomic and metagenomic data provide important information regarding microbial communities and functional potential but do not necessarily reveal which pathways are actively operating or which metabolites mediate host responses.

Future studies should therefore integrate metagenomics with metatranscriptomics, metaproteomics, metabolomics, lipidomics, and host transcriptomics. Such multi-omics approaches may help connect microbial genes with gene expression, protein activity, metabolite production, and host physiological responses. For example, integrating microbial metagenomics with SCFA profiling, bile acid metabolomics, nitrogen metabolism, and host gene expression may clarify whether specific microbial pathways contribute directly to nutrient utilization, intestinal barrier function, immune regulation, or production performance.

However, multi-omics integration should not become an end in itself. Large datasets require appropriate experimental design and biological validation. Candidate microbial pathways identified through multi-omics analyses should be tested using controlled dietary or microbial interventions, targeted metabolite supplementation or inhibition, microbial isolation, and functional assays. The ultimate goal should be to establish mechanistic links between microbial activity and measurable host outcomes.

### Reproducibility and environmental confounding

Reproducibility remains a major challenge in poultry microbiome research. Differences in animal breed, species, age, diet, sampling site, housing system, geographical location, environmental exposure, DNA extraction methods, sequencing platforms, and bioinformatic pipelines can substantially influence microbial profiles.

Environmental confounding is particularly important in poultry because birds are exposed to complex microbial communities from feed, water, litter, housing surfaces, and farm personnel. Geographic location and farm management may therefore influence microbial colonization independently of dietary interventions. Similarly, sampling the ileum, ceca, or feces may produce substantially different microbial profiles because of spatial heterogeneity along the gastrointestinal tract.

Future studies should therefore adopt standardized sampling procedures, clearly defined metadata, appropriate negative controls, consistent sequencing and bioinformatic workflows, and transparent reporting of experimental conditions. Multi-center and multi-location studies will also be valuable for determining whether candidate microbial biomarkers remain stable across production environments.

Importantly, standardization should not eliminate biological diversity. Instead, standardized methodological frameworks should be combined with appropriate statistical models that account for species, age, genotype, diet, environment, and production system. Such approaches may distinguish reproducible biological signals from context-specific microbial responses.

### Species- and age-specific precision microbiome modulation

The concept of a universal "optimal microbiota" is unlikely to be biologically realistic. Broilers, laying hens, ducks, geese, and turkeys differ in digestive physiology, nutrient requirements, growth trajectories, immune responses, and production objectives (Scanes, 2007). Similarly, microbial functions that are beneficial during early intestinal development may not be equally relevant during rapid growth or prolonged egg production (Ballou, et al., 2016).

Future microbiota-based interventions should therefore be designed according to poultry species, developmental stage, production objective, and environmental context. Early-life interventions may focus on establishing a stable microbial ecosystem and supporting intestinal maturation, whereas interventions in growing birds may prioritize nutrient utilization and feed efficiency. In laying hens, long-term intestinal stability and nutrient absorption may be more relevant to sustained egg production and shell quality.

This precision approach should also consider individual variation in baseline microbiota. Birds receiving the same intervention may respond differently because of differences in resident microbial communities, host genotype, immune status, and dietary history. Therefore, future precision nutrition strategies may require microbiome-informed stratification of animals or production systems rather than uniform interventions.

### Artificial intelligence and predictive microbiome

The increasing availability of microbiome, metabolomics, host omics, and production data provides opportunities for artificial intelligence (AI) and machine learning (ML) to identify complex relationships that may not be captured by conventional statistical approaches. Predictive models could potentially identify microbial signatures associated with feed efficiency, disease susceptibility, heat-stress resilience, or response to nutritional interventions.

However, the application of AI and ML to poultry microbiome research remains constrained by limited sample sizes, high-dimensional data, methodological heterogeneity, and insufficient external validation. Models trained on one farm or production system may perform poorly when applied to different populations because microbial signatures are strongly influenced by environmental and management conditions.

Future predictive microbiome models should therefore prioritize biologically interpretable features, external validation, longitudinal datasets, and multi-farm testing. Integrating microbial functional data with host genotype, dietary composition, environmental parameters, and production records may improve predictive accuracy and practical utility. Importantly, AI-based predictions should ultimately be validated through controlled interventions rather than being interpreted as evidence of biological causality.

### Commercial translation, economic feasibility and sustainability

The successful translation of microbiota-targeted strategies from experimental studies into commercial poultry production requires robust evidence demonstrating their reproducibility, scalability, safety, and economic benefits. Although many microbiota-based interventions have shown promising effects under controlled experimental conditions, their performance in commercial production systems is often variable due to differences in diet composition, host genetics, environmental conditions, pathogen exposure, stocking density, management practices, and baseline microbial communities. Therefore, the effectiveness of these strategies should be validated under diverse and representative production environments before widespread adoption.

Commercial evaluation of microbiota-targeted approaches should extend beyond microbial and physiological endpoints and incorporate comprehensive production, economic, and environmental outcomes. Key indicators should include feed conversion ratio (FCR), feed cost per unit of product, body weight gain, egg mass production, mortality, medication use, carcass yield, and meat quality. In addition, environmental benefits, including improved nitrogen utilization, reduced ammonia emissions, and decreased waste generation, should be considered when assessing the overall sustainability of microbiota-based interventions.

Emerging microbiota-targeted technologies face additional challenges during commercialization. Fecal microbiota transplantation (FMT) requires rigorous donor selection, pathogen screening, microbial community standardization, batch-to-batch consistency, and regulatory oversight. Bacteriophage-based approaches are limited by host specificity, phage resistance, formulation stability, and challenges associated with large-scale manufacturing and delivery. Similarly, IgA-based microbiota modulation strategies remain mechanistically promising but require further optimization regarding production efficiency, delivery systems, stability, cost-effectiveness, and regulatory approval. These emerging technologies should therefore be evaluated using integrated frameworks that consider biosafety, regulatory compliance, manufacturing consistency, scalability, economic costs, and stability under commercial farm conditions.

For nutritional microbiota-targeted interventions, additional considerations include feed manufacturing compatibility, product shelf life, and functional stability during storage and processing. Probiotic and synbiotic products must maintain viable and functional microbial strains throughout feed production and distribution, whereas the efficacy of enzymes and prebiotics may depend on feed composition and manufacturing conditions. Consequently, future research should move beyond short-term laboratory trials and conduct standardized, adequately powered, multi-location, and production-scale studies to determine the consistency and practical value of these interventions.

The most realistic pathway toward commercial application is likely to involve integrating microbiota-targeted strategies with existing poultry health management programs rather than relying on individual microbial interventions alone. Optimized nutrition, vaccination, biosecurity, environmental management, and microbiota modulation should be considered complementary components of precision and sustainable poultry production systems. Ultimately, comprehensive assessments combining microbiological, physiological, economic, and environmental outcomes will be essential for determining whether microbiota-targeted strategies can provide reliable and economically meaningful benefits under commercial production conditions.

### Future directions: from descriptive microbiomes to functional precision production

The future of poultry microbiome research should move from cataloging microbial taxa toward understanding and manipulating microbial functions. This transition will require integration of microbial ecology with host genotype, dietary composition, developmental stage, environmental conditions, and production management.

Several priorities should be emphasized. First, longitudinal and intervention-based studies are needed to distinguish transient microbial associations from stable causal mechanisms. Second, multi-omics approaches should be combined with targeted functional validation to identify microbial pathways and metabolites that directly influence host physiology. Third, species- and age-specific precision nutrition strategies should be developed rather than assuming that a single microbiota-based intervention will be effective across all poultry production systems.

Fourth, candidate interventions should be evaluated under commercial conditions using standardized production, economic, environmental, and welfare indicators. Finally, predictive modeling and AI may help identify microbial and host features that can guide individualized or farm-specific interventions, provided that models are externally validated and biologically interpretable.

**Fig. 3:**
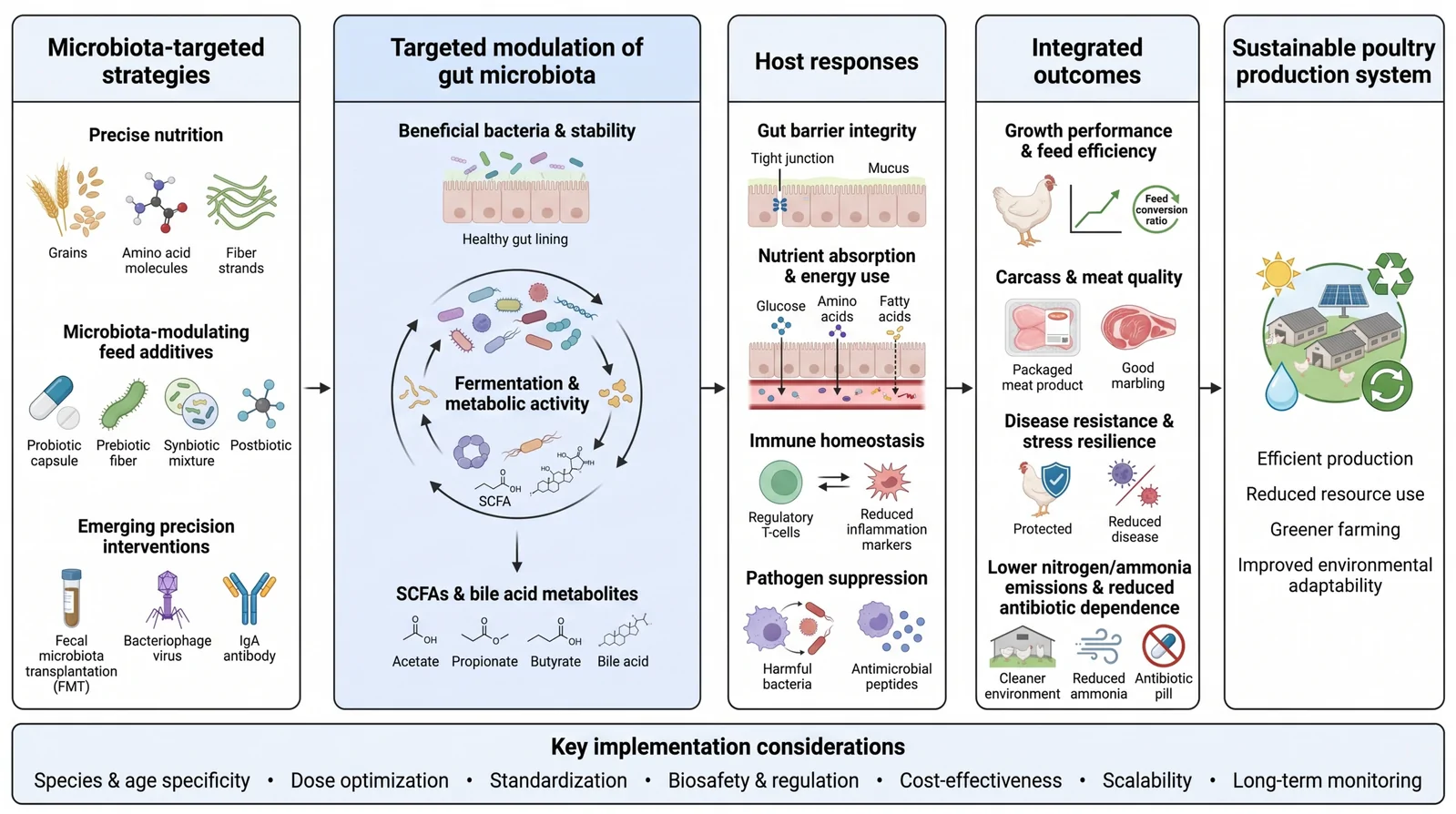
**Integrated framework for microbiota-targeted strategies in sustainable poultry production.** Precision dietary formulation and microbiota-targeted interventions, including probiotics, prebiotics, synbiotics, postbiotics, fecal microbiota transplantation, bacteriophage-based modulation, and IgA-mediated microbiota regulation, provide complementary approaches for manipulating the poultry gut microbial ecosystem. These strategies may alter microbial composition and functional activity, promote beneficial metabolite production, improve intestinal barrier integrity and immune homeostasis, enhance nutrient absorption and energy utilization, and strengthen resistance to pathogens and environmental stress. By improving feed efficiency, growth performance, nitrogen utilization, and animal health, microbiota-targeted strategies may contribute to reduced antimicrobial dependence, lower nitrogen excretion and ammonia emissions, improved animal welfare, and more sustainable poultry production. However, successful translation requires consideration of species- and age-specific responses, intervention efficacy, biosafety, regulatory requirements, reproducibility, scalability, and economic feasibility.

Ultimately, successful application of gut microbiota research in poultry production will depend not on identifying a universal “ideal microbiota,” but on developing reproducible methods to manipulate microbial functions in ways that are biologically effective, economically feasible, environmentally sustainable, and stable under commercial conditions. Integrating precision nutrition, microbiome science, host physiology, AI-based prediction, and farm-level management may provide a foundation for next-generation poultry production systems that simultaneously improve productivity, animal health, welfare, and environmental sustainability (Fig. 3).

## Conclusion

The gut microbiota represents an essential component of poultry physiology and contributes to multiple biological processes, including nutrient metabolism, intestinal barrier maintenance, immune regulation, and production performance (Azizi, et al., 2026). Through the fermentation of indigestible dietary substrates, transformation of amino acids and bile acids, and production of bioactive microbial metabolites, the gut microbiota influences host energy metabolism, intestinal homeostasis, and physiological adaptation. These microbial functions are associated with important production traits, including feed efficiency, growth performance, nutrient utilization, carcass characteristics, meat quality, environmental stress resilience, and nitrogen metabolism (Oakley, et al., 2014b). However, the effects of gut microbiota are highly context-dependent and are shaped by complex interactions among poultry species, developmental stage, dietary composition, host genetic background, and environmental conditions (Pan, et al., 2014).

Nutritional modulation of the gut microbiota, including probiotics, prebiotics, synbiotics, dietary fiber optimization, exogenous enzymes, precision protein nutrition, and emerging microbiota-targeted approaches, provides valuable opportunities to improve intestinal health, optimize nutrient utilization, and support sustainable poultry production while reducing dependence on antibiotic growth promoters (Yadav, et al., 2019). However, variable responses among studies indicate that microbial composition alone is insufficient to predict functional outcomes. A comprehensive understanding of microbial metabolic capacity, functional pathways, and host–microbiota interactions is therefore essential. Importantly, microbial signatures associated with favorable production traits should be interpreted cautiously, as associations between microbial composition and performance do not necessarily demonstrate causal relationships without functional validation.

Overall, current evidence indicates that the value of poultry gut microbiome research lies not in identifying a universal beneficial microbiota, but in understanding how microbial functions interact with host physiology under specific dietary, developmental, genetic, and environmental contexts. Integrating microbiota-targeted nutritional strategies with multi-omics approaches, functional validation, and precision management frameworks will facilitate the development of more efficient, resilient, welfare-oriented, and sustainable poultry production systems. Ultimately, the successful application of microbiome research will depend on the ability to reproducibly optimize microbial functions and host–microbiota interactions rather than simply modifying microbial composition.

## Funding

This work was supported by the Shaanxi Provincial Department of Science and Technology (2023KXJ-243), the Xianyang Science and Technology Bureau (L2024-ZDKJ-ZDCGZH-0007), the China Animal Husbandry Group (ZM202401), and the Shaanxi Engineering Research Center of Lueyang Black Chicken.

## Author Contribution

**Zhuting Chen** and **Cheng Xin** contributed to the conception and design of the study, the acquisition, analysis, and interpretation of the data, and the drafting of the manuscript. **Shuang Zhang** and **Wanyue Meng** contributed to the acquisition, analysis, and interpretation of the data and critically revised the manuscript for important intellectual content. **Deming Zhang** contributed to the analysis and interpretation of the data and critically revised the manuscript for important intellectual content. **Fei Zhang, Qinyi Zhan, Yanli Liu**, and **Xin Yang** contributed to the interpretation of the data and critically revised the manuscript for important intellectual content. **Xiaojun Yang** and **Zhouzheng Ren** contributed to the conception and design of the study, provided financial support, supervised the research, and critically revised the manuscript for important intellectual content. All authors reviewed and explicitly approved the exact final version of the manuscript before publication and agree to be personally and publicly accountable for all aspects of the work, including ensuring that any questions regarding the accuracy or integrity of the research are thoroughly investigated and resolved.

## Disclosures

We declare that we have no financial and personal relationships with other people or organizations that can inappropriately influence our work, and there is no professional or other personal interest of any nature or kind in any product, service and/or company that could be construed as influencing the content of this paper.

## Data Availability

All the data are in the manuscript.
